# NovelmiRNA-25 inhibits AMPD2 in peripheral blood mononuclear cells of patients with systemic lupus erythematosus and represents a promising novel biomarker

**DOI:** 10.1186/s12967-018-1739-5

**Published:** 2018-12-22

**Authors:** Gangqiang Guo, Huijing Wang, Xinyu Shi, Lele Ye, Kai Wu, Kangmin Lin, Sisi Ye, Baoqing Li, Huidi Zhang, Qiaoai Lin, Shuang Ye, Xiangyang Xue, Chaosheng Chen

**Affiliations:** 1 Department of Microbiology and Immunology, Institute of Molecular Virology and Immunology, Institute of Tropical Medicine, School of Basic Medical Sciences, Wenzhou Medical University, Wenzhou, 325035 China; 2 Department of Rheumatology, South Campus, Renji Hospital, Shanghai Jiaotong University School of Medicine, Shanghai, 200025 China; 3 Department of Laboratory Medicine, Second Affiliated Hospital & Yuying Children’s Hospital, Wenzhou Medical University, Wenzhou, 325035 China; 4 Department of Nephrology, First Affiliated Hospital, Wenzhou Medical University, Wenzhou, 325035 China

**Keywords:** RNA-sequencing, SLE, microRNA, AMPD2, Biomarker

## Abstract

**Background:**

Systemic lupus erythematosus (SLE) is a multisystemic autoimmune disease with various clinical manifestations. MicroRNAs (miRNAs) and immunometabolism are recognized as key elements in SLE pathogenesis; however, the relationship between miRNAs in peripheral blood mononuclear cells (PBMCs) and metabolism in SLE remains unclear.

**Methods:**

We detected PBMC miRNA and mRNA profiles from 3 pooled SLE patients and 3 healthy controls (HCs) using next-generation sequencing, predicted miRNA targets in dysregulated mRNAs, predicted functions and interactions of differentially expressed genes using bioinformatics analysis, validated candidate miRNAs using qRT-PCR, and investigated the association between the expression of candidate miRNAs and SLE clinical characteristics. Moreover, we validated the direct and transcriptional regulatory effect of NovelmiRNA-25 on adenosine monophosphate deaminase 2 (*AMPD2*) using a dual-luciferase reporter assay and western blot and confirmed *AMPD2* mRNA and protein expression in PBMCs using qRT-PCR and western blot, respectively.

**Results:**

Multilayer integrative analysis of microRNA and mRNA regulation showed that 10 miRNAs were down-regulated and 19 miRNAs were up-regulated in SLE patient PBMCs compared with HCs. Bioinformatics analysis of regulatory networks between miRNAs and mRNAs showed that 19 miRNAs were related to metabolic processes. Two candidate miRNAs, NovelmiRNA-25 and miR-1273h-5p, which were significantly increased in the PBMCs of SLE patients (P < 0.05), represented diagnostic biomarkers with sensitivities of 94.74% and 89.47%, respectively (area under the curve = 0.574 and 0.788, respectively). NovelmiRNA-25 expression in PBMCs was associated with disease activity in SLE patients, in both active and stable groups (P < 0.05). NovelmiRNA-25 overexpression downregulated *AMPD2* expression in HEK293T cells through direct targeting of the *AMPD2* 3ʹUTR (P < 0.01), while inhibition of NovelmiRNA-25 activity led to increased *AMPD2* expression (P < 0.01). NovelmiRNA-25 overexpression also downregulated AMPD2 protein expression in HEK293T cells; AMPD2 protein expression in SLE patient PBMCs was decreased. Our results show that differentially expressed miRNAs play an important role in SLE.

**Conclusions:**

Our data demonstrate a novel mechanism in SLE development that involves the targeting of *AMPD2* expression by NovelmiRNA-25. miRNAs may serve as novel biomarkers for the diagnosis and evaluation of disease activity of SLE and represent potential therapeutic targets for this disease.

**Electronic supplementary material:**

The online version of this article (10.1186/s12967-018-1739-5) contains supplementary material, which is available to authorized users.

## Background

Systemic lupus erythematosus (SLE), an autoimmune disease mediated by pathogenic autoantibodies, presents with severe clinical manifestations such as nephritis, multisystem organ failure, or central nervous system disease [1]. Prevalence rates of SLE worldwide vary from approximately 20 to 70 per 100,000 person-years, and incidence rates for women are approximately ten times higher than those for men [2]. As the pathogenesis of SLE remains unclear, treatment, which mainly consists of glucocorticoid-antimalarial drugs and non-steroidal anti-inflammatory drugs (NSAIDs), often focuses on immune suppression to prevent and control disease progression, but flare still occurs [3, 4]. Therefore, the mortality rate of SLE remains high owing to the severity of the disease and the resulting organ damage [1].

Accumulating evidence has indicated that metabolic syndrome is a major co-morbidity of SLE [5]. Of note, there is a growing understanding that cellular metabolic processes result in changes in inflammatory and immune responses in various immune cell populations [6], including macrophages, dendritic cells, neutrophils, and peripheral blood mononuclear cells (PBMCs) such as CD4^+^ T cells and B cells [7–9]. Specific metabolic processes are critical checkpoints of effector functions in the immune system, with common as well as cell-specific programs [7]. Lupus-prone mice and SLE patients have been found to exhibit activated metabolism of CD4^+^ T cells; the use of metabolic inhibitors was found to effectively normalize these features and was associated with therapeutic effects [10]. Furthermore, a proof-of-concept trial found a reduction in clinical flare-ups following metformin add-on treatment in mild or moderate SLE [11], thereby demonstrating that drugs involved in metabolic regulation may be used as add-on treatment in autoimmune diseases.

MicroRNAs (miRNAs) are small, noncoding RNAs that regulate numerous immunologic, inflammatory, and oncogenic pathways by modulating protein translation [12, 13]. miRNAs have been shown to play crucial roles in SLE pathogenesis [14], particularly lupus susceptibility, through their fundamental mechanism of post-transcriptional regulation of gene expression, suggesting their potential utility as diagnostic markers or therapeutic targets in this disease [15]. Dysregulation of miRNAs has been associated with disease activity and major organ involvement in patients with SLE [16]. For example, compared with that in healthy individuals, the expression of miRNA miR-21 is elevated in CD4^+^ T cells from lupus patients; overexpression of miR-21 suppressed the expression of PDCD4, a selective protein translation inhibitor, and regulated aberrant T cell responses [17]. However, data on the relationship between miRNAs in PBMCs and metabolism in SLE has not yet been reported.

In this study, we first evaluated mRNA and miRNA expression profiles in PBMCs from Chinese SLE patients using next-generation sequencing (NGS). Secondly, integrating the results of our bioinformatic analysis of immunometabolism, we sought to identify potential miRNAs associated with the regulation of SLE progression and experimentally validated their differential expression between SLE patients and healthy controls (HCs). Lastly, through dual-luciferase reporter assays and western blotting, we identified the potential metabolism-associated targets that are regulated by miRNAs. The purpose of this study was to elucidate the mechanisms underlying SLE progression and investigate the potential applications of miRNAs in the treatment of this disease.

## Methods

### Subjects and study design

All 28 SLE patients admitted to the Department of Rheumatology and Nephrology, First and Second Affiliated Hospital of Wenzhou Medical University, between June 2016 and January 2018, were enrolled in this study. They were divided into two main independent SLE cohorts. The primary exploratory cohort consisted of three female patients, and the validation cohort comprised 25 patients. Moreover, PBMCs from 5 SLE patients and 5 HCs were selected randomly to validate the protein expression of miRNA target genes. All patients fulfilled the American College of Rheumatology (ACR) 1997 criteria for SLE [18]. Disease activity was assessed according to the Systemic Lupus Erythematosus Disease Activity Index (SLEDAI) [19] at the time of blood collection. SLE patients with SLEDAI ≥ 5 were defined as having active disease, and those with SLEDAI < 5 were defined as having stable disease. The characteristics of all subjects are summarized in Additional file 3: Table S1. Twenty-eight age- and sex-matched healthy controls (HCs) without arthralgia, heart failure, renal failure, or autoimmune disease, and free from other inflammatory conditions, were recruited based on records available from the Wenzhou local blood bank. The research protocol was approved by the Medical Ethical Committees of the First and Second Affiliated Hospital of Wenzhou Medical University. All subjects who participated in this research provided written informed consent.

### PBMC and RNA isolation

PBMCs were isolated from SLE patients and HCs using human peripheral blood lymphocyte separation medium (Tianjin Hao Yang Biological Manufacture, Tianjin, China) within 4 h of collection of the samples. Total RNA was extracted from each sample using TriZol Reagent (Invitrogen Life Technologies^®^, Grand Island, NY, USA). The isolated RNAs were digested by Dnase I (Invitrogen™, Waltham, MA, USA) to remove the residual DNA, and were then collected in 25 μL of DNase/RNase-free water. The concentration of RNA was quantified and qualified using a NanoDrop instrument (Thermo Fisher Scientific Inc., Waltham, MA, USA) and 1% agarose gel electrophoresis. Isolated RNA was stored at − 80 °C for use. The samples from the exploratory cohort were used for NGS analysis. Samples from the validation cohort were used for validation by quantitative reverse-transcription (qRT)-PCR.

### NGS profiling

All protocols, including NGS library preparation, were conducted according to the manufacturer’s instructions l (NEBNext^®^ Ultra™ Directional RNA Library Prep Kit for Illumina^®^). Genewiz Ltd (https://www.genewiz.com.cn/) was used for all steps, from RNA library preparation to the generation of alignment files [20].

For small RNA sequencing, 3′ SR Adaptors for Illumina were ligated to small RNAs using the 3′ Ligation Enzyme. To prevent adaptor-dimer formation, excess 3′ SR Adaptors were hybridized with SR RT Primer for Illumina. Then, 5′ SR Adaptors for Illumina were ligated to the small RNAs using 5′ Ligation Enzyme, and first-strand cDNA was synthesized using ProtoScript II Reverse Transcriptase. Each sample was then amplified by PCR for 12 cycles using P5 and P7 primers, whose sequences annealed to flow cells to allow bridge PCR to be performed. Further, the P7 primer carried a six-base index allowing for multiplexing. PCR products of ~ 140 bp were recovered and cleaned up using PAGE, validated using an Agilent 2100 Bioanalyzer (Agilent Technologies, Palo Alto, CA, USA), and quantified using a Qubit 2.0 Fluorometer (Invitrogen, Carlsbad, CA, USA). Then, libraries with different indices were multiplexed and loaded on an Illumina HiSeq instrument according to the manufacturer’s instructions (Illumina, San Diego, CA, USA). Sequencing was carried out using a 1 × 50 bp single-end (SE) configuration; image analysis and base calling were conducted with HiSeq Control Software (HCS) + OLB + GAPipeline-1.6 (Illumina) on the HiSeq instrument.

For transcriptome sequencing, the poly(A) mRNA isolation was performed using NEBNext Poly(A) mRNA Magnetic Isolation Module (NEB). mRNA fragmentation and priming were performed using NEBNext First Strand Synthesis Reaction Buffer and NEBNext Random Primers. cDNAs were synthesized, purified, treated, amplified, validated, and quantified, respectively. Then, libraries with different indices were multiplexed and loaded on an Illumina HiSeq 2000 instrument according to the manufacturer’s instructions. Sequencing was carried out using a 2 × 150 bp paired-end (PE) configuration; image analysis and base calling were conducted with HiSeq Control Software (HCS) + OLB + GAPipeline-1.6 (Illumina) on the HiSeq instrument.

RNA-seq results were converted into read count values using Bioconductor Software DESeq (v.1.6.3) or edgeR (v.3.4.6) [21]. Transcript expression levels were calculated using fragments per kilobase of transcript per million fragments mapped (FPKM) values. Fold changes in RNA expression between SLE patients and HCs were calculated from the signal values. Differential expression of miRNAs was considered significant at a fold change > 2 and P-value ≤ 0.05. Candidate circulating miRNAs were chosen for subsequent analysis based on a fold change > 2 and potential for association with target mRNAs. Similar to miRNAs, differential mRNA expression was considered significant at a fold change > 2 and false discovery rate (FDR) ≤ 0.05.

### GO and KEGG pathway analysis

The functions of all differentially expressed miRNAs and mRNAs were investigated using Gene Ontology (GO) annotations and Kyoto Encyclopedia of Genes and Genome (KEGG) pathway analysis. Hierarchical clustering of the differentially expressed genes (DEGs) according to the biological process (BP), cellular component (CC), and molecular function (MF) categories was performed by GO analysis to elucidate genetic regulatory networks (http://www.geneontology.org). Pathway analysis using graphical diagrams was performed to explore DEG pathways using the KEGG database (http://www.genome.jp/kegg/). Significance was determined by P-value and FDR.

### Target gene prediction and bioinformatics analysis of miRNAs and mRNAs

Based on the miRNA sequences and mRNA expression profiles in the exploratory cohort, potential targets sites for candidate miRNAs were predicted using the miRanda software (https://www.genewiz.com.cn/). Then, miRNAs were further filtered by combining prediction of miRNA-target pairs with mRNA profiling data. Subsequently, the functions of potential miRNAs were investigated by GO analysis using PANTHER software (http://www.pantherdb.org). By integrating the miRNA-mRNA regulatory network, a miRNA-gene regulatory network was constructed. To further explore the biological functions and build a network of the functions of miRNA target genes in SLE, the Thomson Reuters database (https://portal.genego.com) [22] was used. A coexpression network was constructed for the 29 candidate miRNAs and target genes using Cytoscape software. In addition, RNAhybrid software [23] was used to predict the resulting secondary structures of mRNA and miRNA interactions.

To identify miRNAs that play important roles in SLE development, the following criteria were applied (1) the key miRNAs and mRNAs should show significant expression changes between SLE patients and HCs; (2) they should exhibit a detectable negative relationship between the expression of the miRNA and the mRNA target genes; (3) the miRNA target genes should be associated with metabolic process in the BP category; (4) the binding energy between the miRNA and mRNA should be < − 31 kcal/mol (Table 1, Additional file 3: Table S7); (5) the 2–8 bp of the miRNA seed region should be strictly matched with target genes; (6) the expression level of target genes of miRNA should be log2foldchange > |2.9|.

**Table 1:** Interaction of miRNA and mRNA in SLE patients

miRNA_ID	miRNA_log_2_^FC^	miRNA_Regulation	Target mRNA_ID	Target mRNA gene symbol	Target mRNA_log_2_^FC^	Target mRNA_Regulation
all_hsa-miR-1260a	NA^#^	Down	ENST00000367696	*RC3H1*	2.162	Up
NovelmiRNA-72	NA	Down	ENST00000361776	*FOXJ3*	9.087	Up
			ENST00000369248	*FAM160B1*	Inf	
			ENST00000335515	*PRICKLE4*	Inf	
			ENST00000431061	*ELL2*	5.190	
			ENST00000314330	*C9orf139*	2.595	
			ENST00000436936	*NLRC5*	Inf	
			ENST00000426335	*ARFGAP2*	5.775	
			ENST00000315588	*MED29*	Inf	
			ENST00000531165	*CELF1*	Inf	
			ENST00000532415	*PKD1P5*	4.679	
			ENST00000578036	*PLEKHM1P*	Inf	
all_hsa-miR-4470	Inf*	Up	ENST00000406337	*KAT6A*	− 2.213	Down
NovelmiRNA-798	Inf	Up	ENST00000374282	*PAFAH2*	− 6.846	Down
all_hsa-miR-150-5p	− 1.523	Down	ENST00000304363	*SUV420H1*	Inf	Up
			ENST00000354670	*BMF*	Inf	
NovelmiRNA-376	NA	Down	ENST00000335934	*TP53I3*	4.654	Up
			ENST00000394519	*RNF14*	4.642	
			ENST00000354670	*BMF*	Inf	
			ENST00000254654	*ILKAP*	Inf	
all_hsa-miR-330-5p	1.469	Up	ENST00000438423	*TCF12*	− 1.990	Down
			ENST00000442101	*ELF1*	− 2.653	
			ENST00000518421	*AP3M2*	− 2.599	
			ENST00000566433	*ADCY7*	− 4.518	
all_hsa-miR-874-5p	− 1.713	Down	ENST00000360121	*SPN*	2.820	Up
			ENST00000535612	*ARMC6*	Inf	
			ENST00000316509	*VAMP2*	Inf	
			ENST00000335515	*PRICKLE4*	Inf	
			ENST00000588731	*SUGP1*	3.932	
			ENST00000354329	*MYO18A*	Inf	
			ENST00000388995	*FAM83G*	Inf	
			ENST00000528896	*KIAA0100*	Inf	
			ENST00000242351	*ZC3HAV1*	Inf	
			ENST00000378387	*ARHGEF39*	4.053	
			ENST00000313028	*PARP10*	Inf	
			ENST00000495880	*DUSP7*	2.318	
			ENST00000449969	*PLCH2*	Inf	
			ENST00000562522	*ITGAX*	Inf	
			ENST00000492450	*ZNF181*	Inf	
			ENST00000442312	*SH3BP2*	Inf	
			ENST00000314330	*C9orf139*	2.595	
			ENST00000453547	*ARHGAP19*-*SLIT1*	5.478	
			ENST00000311895	*ERCC4*	Inf	
			ENST00000397527	*CEP250*	3.423	
			ENST00000436936	*NLRC5*	Inf	
			ENST00000325722	*KIAA0319L*	4.037	
			ENST00000251582	*ADAMTS2*	9.369	
			ENST00000447018	*CCDC51*	5.414	
			ENST00000373130	*STK40*	Inf	
			ENST00000589297	*CYTH1*	5.446	
			ENST00000374134	*RGS3*	2.658	
			ENST00000578036	*PLEKHM1P*	Inf	
all_hsa-miR-342-5p	− 1.303	Down	ENST00000356187	*RNH1*	Inf	Up
all_hsa-miR-106b-3p	Inf	Up	ENST00000295760	*FBLN2*	-5.518	Down
NovelmiRNA-815	NA	Down	ENST00000378387	*ARHGEF39*	4.053	Up
			ENST00000347230	*ZFYVE26*	Inf	
			ENST00000357867	*FN1*	Inf	
			ENST00000315588	*MED29*	Inf	
			ENST00000375759	*SPEN*	Inf	
			ENST00000450305	*DDX11L1*	Inf	
NovelmiRNA-288	NA	Down	ENST00000449969	*PLCH2*	Inf	Up
			ENST00000453547	*ARHGAP19*-*SLIT1*	5.478	
			ENST00000346169	*EIF4G1*	2.275	
all_hsa-miR-7854-3p	1.704	Up	ENST00000310826	*ZBTB21*	− 4.660	Down
			ENST00000331222	*CLN8*	NA	
			ENST00000220597	*PAG1*	− 2.120	
			ENST00000426496	*PRRC2C*	− 3.820	
NovelmiRNA-983	Inf	Up	ENST00000416476	*BBX*	− 2.929	Down
NovelmiRNA-489	Inf	Up	ENST00000339562	*NR4A2*	− 2.229	Down
			ENST00000570054	*RP11*-*343C2.11*	NA	
NovelmiRNA-862	NA	Down	ENST00000348428	*MALT1*	7.635	Up
			ENST00000555447	*STON2*	2.385	
			ENST00000450053	*NBEAL2*	6.383	
			ENST00000314330	*C9orf139*	2.595	
			ENST00000264033	*CBL*	4.351	
			ENST00000361842	*XAF1*	2.457	
			ENST00000346169	*EIF4G1*	2.275	
			ENST00000526177	*GDPD5*	5.050	
			ENST00000301202	*LAIR2*	2.836	
NovelmiRNA-30	Inf	Up	ENST00000491381	*SNX21*	− -4.412	Down
			ENST00000398246	*LONRF1*	− 2.309	
			ENST00000597188	*ADAMTS10*	NA	
			ENST00000243077	*LRP1*	NA	
			ENST00000380746	*DNMT3A*	NA	
			ENST00000372269	*FAM78A*	− 3.099	
			ENST00000257247	*AHNAK*	NA	
			ENST00000427718	*FBXO21*	NA	
			ENST00000228862	*DUSP16*	− 3.062	
			ENST00000544627	*ATF7IP*	− 1.915	
			ENST00000409645	*MGAT5*	− 1.996	
all_hsa-miR-3150a-5p	2.297	Up	ENST00000525038	*C11orf30*	NA	Down
all_hsa-miR-361-5p	− 1.103	Down	ENST00000342624	*TMTC4*	7.110	Up
			ENST00000539778	*CMIP*	3.559	
			ENST00000526177	*GDPD5*	5.050	
			ENST00000375759	*SPEN*	Inf	
			ENST00000267079	*MAP3K12*	Inf	
NovelmiRNA-426	Inf	Up	ENST00000371030	*ZNF831*	− 3.221	Down
			ENST00000251268	*MEGF8*	NA	
			ENST00000304987	*SIK2*	− 2.195	
			ENST00000243077	*LRP1*	NA	
			ENST00000262518	*SRCAP*	− 3.718	
			ENST00000418048	*CSF3R*	NA	Down
all_hsa-miR-1273 h-5p	1.169	Up	ENST00000367080	*PFKFB2*	NA	Down
			ENST00000552810	*CEP290*	− 5.056	
			ENST00000264951	*XRN1*	NA	
			ENST00000589042	*TTN*	− 2.833	
			ENST00000397708	*MCM3AP*	− 2.945	
			ENST00000409993	*DPAGT1*	NA	
			ENST00000544583	*ABR*	NA	
			ENST00000394961	*SMARCAD1*	− 2.347	
			ENST00000358583	*GOLGA6L20*	NA	
			ENST00000536441	*SESN3*	− 3.579	
all_hsa-miR-92b-5p	1.213	Up	ENST00000258886	*IREB2*	− 5.167	Down
NovelmiRNA-92	NA	Down	ENST00000301749	*NLRC3*	Inf	Up
			ENST00000407690	*TFIP11*	3.053	
			ENST00000266564	*PEX5*	Inf	
			ENST00000309955	*CFLAR*	Inf	
			ENST00000296603	*LMBRD2*	3.287	
			ENST00000380743	*SMN2*	3.576	
			ENST00000417961	*SLC9A8*	3.225	
			ENST00000495880	*DUSP7*	2.318	
			ENST00000396832	*CSNK1E*	Inf	
			ENST00000393667	*GOLGB1*	Inf	
			ENST00000344138	*GRAP2*	4.341	
			ENST00000450053	*NBEAL2*	6.383	
			ENST00000314330	*C9orf139*	2.595	
			ENST00000461206	*ST3GAL5*	4.203	
			ENST00000401949	*GRB10*	3.459	
			ENST00000373823	*GSN*	Inf	
			ENST00000393265	*SLC24A4*	2.727	Up
all_hsa-miR-503-5p	1.246	Up	ENST00000595840	*LRRC25*	− 2.304	Down
all_hsa-miR-6716-5p	Inf	Up	ENST00000493114	*IRF4*	− 4.622	Down
NovelmiRNA-296	Inf	Up	ENST00000374152	*ARID1A*	NA	Down
			ENST00000369393	*MDN1*	− 2.000	
NovelmiRNA-49	Inf	Up	ENST00000344028	*CD6*	− 2.367	Down
			ENST00000589042	*TTN*	− 2.833	
			ENST00000418265	*ISY1*-*RAB43*	NA	
NovelmiRNA-25	2.337	Up	ENST00000417379	*AATK*	NA	Down
			ENST00000394378	*SYNRG*	NA	
			ENST00000254605	*RRP8*	NA	
			ENST00000528667	*AMPD2*	− 2.912	
NovelmiRNA-974	Inf	Up	ENST00000450123	*BCL6*	NA	Down
			ENST00000426496	*PRRC2C*	− 3.820	

NA^#^: only expressed in normal controls; Inf*: only expressed in SLE patients

### qRT-PCR validation of identified miRNAs and miRNA target gene

From the 19 potential miRNAs identified in our comprehensive analysis, three representative miRNAs (miR-874-5p, miR-1273h-5p, and NovelmiRNA-25) were selected and validated by qRT-PCR. The miRNA expression levels in the PBMCs from 50 members of the validation cohort were detected using stem-loop qRT-PCR and the mirVana qRT-PCR miRNA Detection Kit (Ambion, Carlsbad, CA, USA). The miRNA target gene expression levels in the PBMCs from SLE patients of the validation cohort were detected using qRT-PCR. All qRT-PCR reactions were carried out on an Applied BioSystems 7500 Real-Time PCR system (Life Technologies). For each reaction, 1 μL of diluted cDNA was mixed with 5 μL of 2× SYBR Green Reaction Mix (DBI, Ludwigshafen, Germany). A final volume of 10 μL was achieved by the addition of 200 nM of forward and reverse primers. The conditions for PCR amplification were as follows: 95 °C for 3 min, followed by 39 cycles of 95 °C for 10 s and 58 °C for 30 s. The fluorescence signal was measured once every 1 °C. The specificity of the primer amplicons was tested by melting curve analysis. All samples were tested in triplicate. The data were analyzed using the comparative threshold cycle (Ct) method. *U6/*18S ribosomal RNA was used as a control, and the relative quantification of miRNAs in PBMCs was calculated using the following equation: Amount of target = 2^−ΔCt^, where ΔCt = Ct _PBMC miRNAs_ − Ct _U6/18S ribosomal RNA_. The gene-specific primers used are listed in Additional file 3: Table S2.

### Cell culture and transfection

HEK293T cells were cultured in Dulbecco’s modified Eagle’s medium (DMEM) supplemented with 10% fetal bovine serum (FBS) at 37 °C in a humidified atmosphere containing 5% CO_2_ (SANYO, MCO-175, Osaka prefecture, Japan). For cell transfection, HEK293T cells were seeded into 24-well plates at a density of 2 × 10^4^ cells/well. Additionally, 60 nM of NovelmiRNA-25 mimic (5′-AGCAGGACGGUGGCCAUGG-3′; Guangzhou RiboBio Co., Ltd., Guangzhou, China) or a negative control (NC) mimic (Guangzhou RiboBio Co., Ltd.) was mixed with 1 µg of pmirGLO, pmirGLO-h_AMPD2 3′UTR, or pmirGLO-h_AMPD2 3′UTR-mut (GenBank: NM_001257360; Shanghai Bio-Link Co., Ltd.) using X-tremegene HP (Roche, Basel, Switzerland; No. 06366236001) according to the manufacturer’s protocols. Following this, the mixture was added to the 24-well plates to obtain a final concentration of 20 µM of NovelmiRNA-25 mimic or NC mimic. Plates were subsequently incubated at 37 °C for 48 h. Twenty-four wells were tested for each treatment to obtain the mean luciferase activity of each experiment. All experiments were performed in triplicate.

### Luciferase reporter assay

Luciferase activity was measured 48 h after transfection using the Dual-Luciferase Reporter Assay System (Promega, Madison, WI, USA; E1910) in a Luminoskan Ascent (BioTek, Synergy HT, Vermont, USA) according to the manufacturer’s protocols. Firefly luciferase activity was normalized to *Renilla* luciferase activity to account for variations in transfection efficiency between experiments.

### Western blotting

Forty-eight hours after NovelmiRNA-25 mimic transfection, HEK293T cells and PBMCs from 5 SLE patients and 5 HCs were lysed using protein lysis buffer (Beyotime Institute of Biotechnology, Beijing, China) supplemented with protease inhibitor cocktail (Pierce, Rockford, IL, USA) at 4 °C for 20 min. Protein samples were separated using 10% sodium dodecyl sulfate (SDS)-polyacrylamide gel electrophoresis and then electrophoretically transferred to polyvinylidene difluoride membranes (Millipore, Billerica, MA, USA). Antibodies against AMPD2 (ab31537, MULTI SCIENCES, Hangzhou, China) were diluted with primary antibody dilution buffer (Beyotime Institute of Biotechnology) at a 1:400 dilution, while those against GAPDH (GOOD HERE, Hangzhou, China) were diluted 1:1000. The membranes were then washed with TBST buffer five times for 5 min each and incubated with horseradish peroxidase (HRP)-conjugated goat anti-rabbit IgG secondary antibody (1:5000 dilution) (MULTI SCIENCES) for 1.5 h at 37 °C. Bands were detected using enhanced chemiluminescence and visualized with a Gel Doc 2000 (BioRad, Hercules, CA, USA).

### Statistical analysis

Statistical analysis was performed with SPSS 22.0 software (SPSS, Inc., Chicago, IL, USA). Data are presented as mean ± standard deviation. Statistical significance between groups was determined by Student’s *t* test or Chi square test. A P-value < 0.05 was considered to represent statistically significant differences. Receiver operating characteristic (ROC) curve analysis, plotting the true positive rate (sensitivity) versus the false positive rate (1 − specificity) at various threshold settings, was performed for PBMC miRNAs, and the areas under curve (AUC) were calculated using Medcalc 15.2.2. The maximum of the sum of true positive rate and false positive rate was calculated, and cutoff value with higher specificity was selected. Expression graphs and ΔCt values were analyzed using GraphPad Prism version 5.04 software (GraphPad Software, La Jolla, CA, USA).

## Results

### Overview of RNA-seq data for SLE patients and healthy controls

In order to investigate miRNA and mRNA interactions, the expression profiles of miRNAs and mRNAs in PBMCs from three SLE patients and three HCs (clinical characteristics of patients are shown in Additional file 3: Table S1) were analyzed. Using the Illumina HiSeq 2000 platform, an RNA-seq dataset of 616 million raw reads was obtained from the six samples (ranging from 92 million to 117 million). Using millions of short reads mapped to the human genome, the expression levels of known genes in each sample were quantified using the conventional FPKM parameter [24]. Among annotated RefSeq coding genes, on average, 26,900 genes per sample exhibited detectable expression (RPKM > 0.05, roughly one mapped read for one 1-kb-exon gene; Additional file 3: Table S3). To evaluate the overall quality of our RNA-seq data, the RNA-seq data from multiple samples were compared using Pearson’s correlation and the Spearman and Kendall-tau rank correlation and found significant coefficients. On average, 91.8% (94.2 million per sample) of the short reads uniquely mapped to the human reference genome (hg19) or exon-junction sequences (Additional file 3: Table S3). No significant differences in the percentage of aligned reads between samples (SLE vs. HC: 90.0% vs. 93.2%) were observed, indicating the reliability of the sequencing results.

The application of the small RNA-seq protocol to these six samples (three SLE and three HCs) resulted in a small RNA-seq dataset of 1.88 million 20–25-nt short reads (mean of 313,000; Additional file 3: Table S3). On average, 48.7% (152,700 per sample) mapped to known miRNAs in the miRBase database [25] (Additional file 3: Table S3). Evaluation of repetitive reads was based on identical read sequences and compared to the corresponding genome location. Further data analysis revealed uniform distribution of the quality of the RNA-seq.

### Analysis of SLE-related differentially expressed miRNAs and mRNAs

**Fig. 1:**
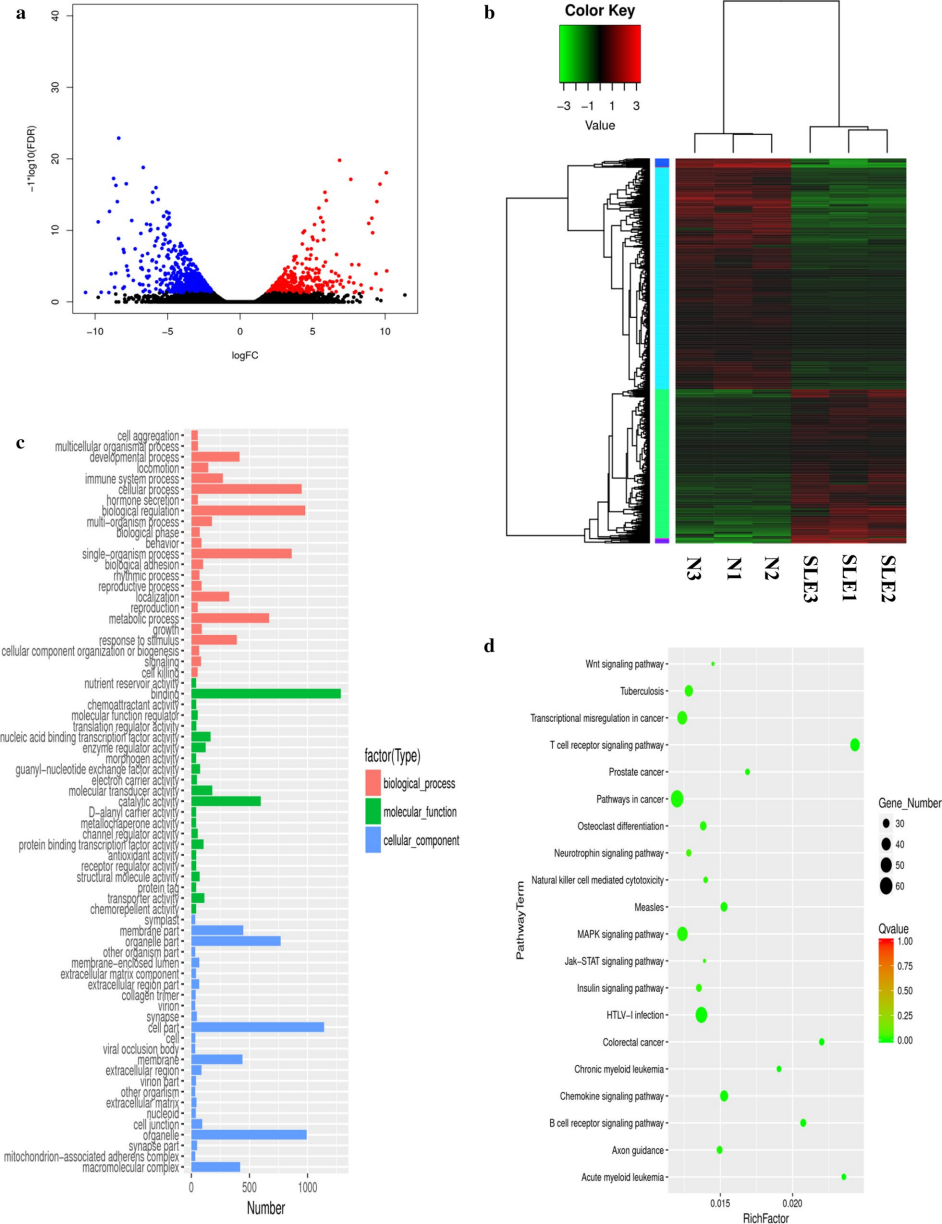
Differential expression of PBMC mRNAs between SLE patients and healthy controls in the exploratory cohort. **a** Volcano plot of differentially expressed mRNAs. The blue spots indicate significantly downregulated mRNAs, and the red spots indicate significantly upregulated mRNAs. **b** Hierarchical clustering of the differentially expressed mRNAs. Blue represents relatively lowly expressed mRNAs, and red represents relatively highly expressed mRNAs. **c** Gene ontology (GO) analysis of differentially expressed mRNAs. Red indicates molecular function (MF), green indicates cellular component (CC), and blue indicates biological process (BP). **d** Pathways of differentially expressed mRNAs. The vertical axis indicates the pathway term, and the horizontal axis indicates the richness factor. The size of the spots represents the number of differentially expressed mRNAs, and the color of the spots represents Q-values ranging from 0 to 1 (the smaller the Q-value, the more significantly enriched the target genes)

To identify SLE-related DEGs, we analyzed the log-scale-transformed RPKM data of protein-coding genes across two cohorts. For mRNA expression profiling, a P-value ≤ 0.05 and FDR ≤ 0.05 [26] were set as threshold values, and 1781 genes were determined to exhibit at least a two-fold difference in expression between SLE-PBMCs and HC-PBMCs. Among these, 713 and 1068 genes were upregulated and downregulated, respectively, in SLE-PBMCs compared with levels in HC-PBMCs. A volcano plot revealed the variance in DEGs (Fig. 1a), and differentially expressed mRNAs were visualized by heat map (Fig. 1b).

**Fig. 2:**
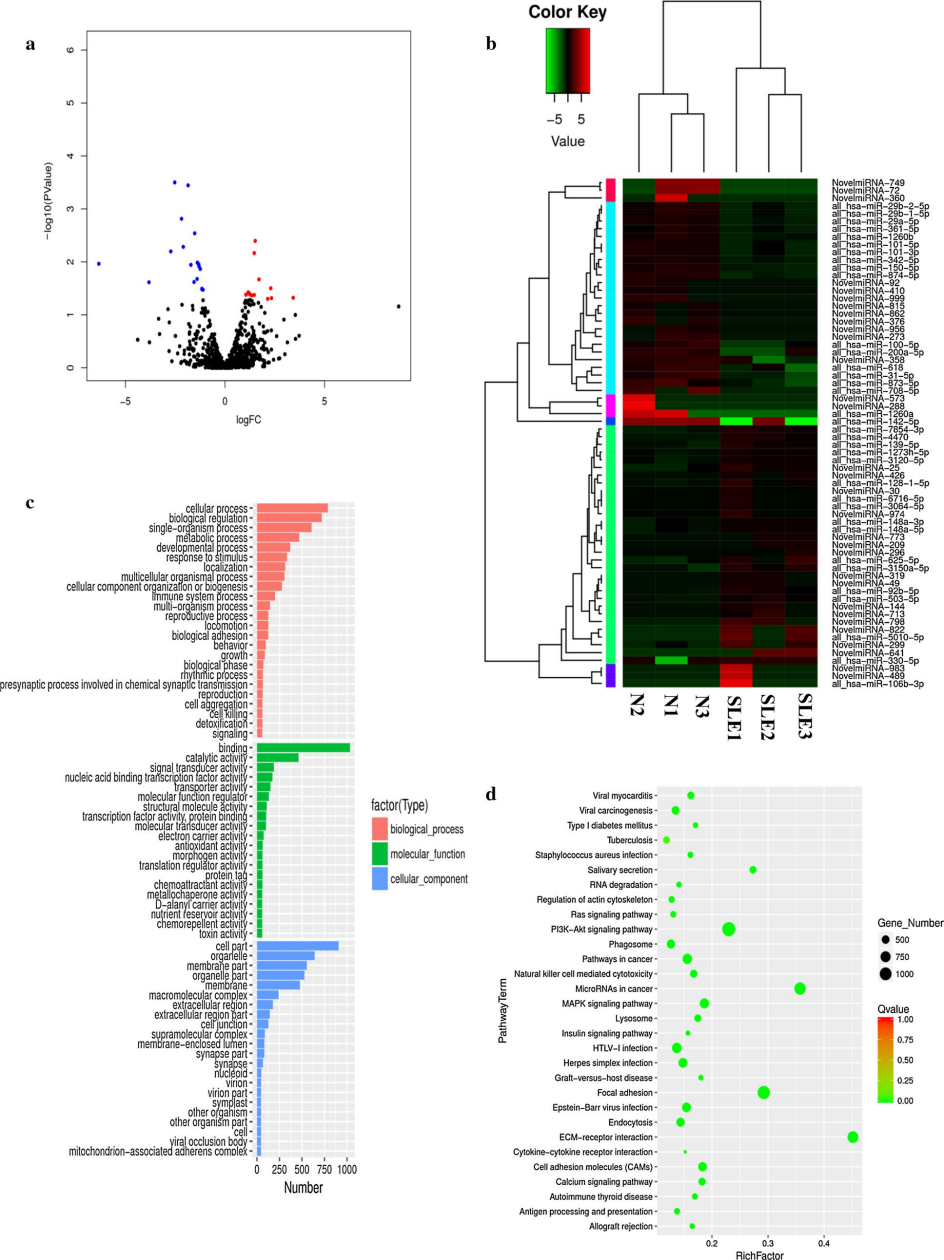
Differential expression of PBMC miRNAs between SLE patients and healthy controls in the exploratory cohort. **a** Volcano plot of differentially expressed miRNAs. The blue spots indicate significantly downregulated miRNAs, and the red spots indicate significantly upregulated miRNAs. **b** Hierarchical clustering of the differentially expressed miRNAs. Red represents relatively highly expressed miRNAs, and blue represents relatively lowly expressed miRNAs. **c** Gene ontology (GO) analysis of differentially expressed miRNAs. Red indicates biological process (BP), green indicates molecular function (MF), and blue indicates cellular component (CC). **d** Pathways of the target genes of differentially expressed miRNAs. The vertical axis indicates the pathway term, and the horizontal axis indicates the richness factor. The size of the spots represents the number of target genes of differentially expressed miRNAs, and the color of the spots represents the Q-value

Based on RPKM values, at a P ≤ 0.05 and fold change > 2 (FDR≤ 0.05), 66 miRNA genes showed significant differential expression: 34 were upregulated and 32 were downregulated in SLE-PBMCs (Fig. 2a, b and Additional file 3: Table S4). Differential expression between miRNAs in the PBMCs of SLE patients and those in HCs was visualized by volcano plot (Fig. 2a) and heat map (Fig. 2b). The fold changes of the significantly differentially expressed miRNAs are listed in Additional file 3: Table S4.

### GO and KEGG pathway analysis

GO analysis showed that differentially expressed mRNAs and miRNAs were enriched in some common biological functions. In the MF category, enriched terms included binding, catalytic activity, and molecular transduction activity. In the BP category, enriched terms included biological regulation, cellular process, and metabolic process, and in the CC category, enriched terms included cell part, organelle part, and macromolecular complex. The GO terms of the differentially expressed mRNAs and miRNAs are shown in Figs. 1c and 2c, respectively.

The cellular processes of miRNA targets were further analyzed using the Thomson Reuters database. The results show that differentially expressed miRNAs were enriched for regulatory roles in various cellular and metabolic processes, as well as cell development and differentiation (Additional file 2A and Additional file 3: Table S5). Most relevant networks of differentially expressed miRNAs involved the movement of cell or subcellular component (91.5%) or cell morphogenesis (76.6%) as the top-scored network and cell adhesion (42.0%) or biological adhesion (42.0%) as the second-scored network (Additional file 2B and Additional file 3: Table S6). These results indicate that the differentially expressed mRNAs and target genes of differentially expressed miRNAs are involved in similar biological functions.

Correspondingly, the molecular function pathways were analyzed using KEGG pathway analysis of differentially expressed mRNAs and target genes of differentially expressed miRNAs (Figs. 1d and 2d). Differentially expressed mRNAs and target genes of differentially expressed miRNAs were both enriched in pathways such as the MAPK signaling pathway, NK cell-mediated cytotoxicity, and other receptor and chemokine signaling pathways. The molecular function of the associated MAPK signaling pathway involves regulation of cell differentiation and cellular metabolism [5]. These functional analyses therefore identified relevant metabolic and cellular processes that are important in the development of autoimmune diseases.

### Interactions of key differentially expressed mRNAs and miRNAs related to SLE

**Fig. 3:**
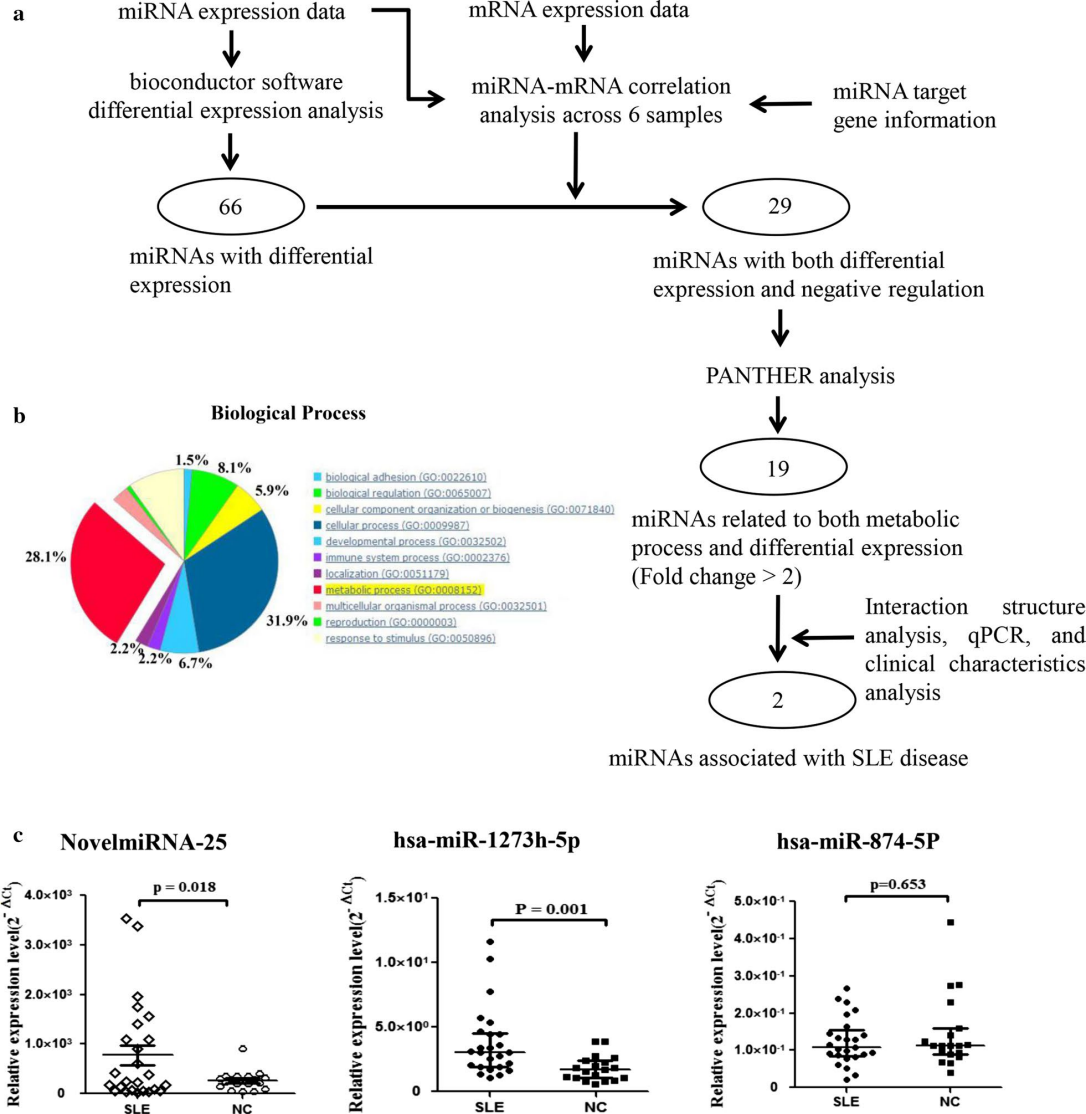
Construction of miRNA-target gene regulatory network and bioinformatic analysis. **a** Data analysis overview. miRNA and mRNA expression data were analyzed by Bioconductor for differential expression (fold change > 2 and P < 0.05). Combining these data and miRNA-target gene data, 29 miRNAs exhibiting both differential expression and negative regulation of target genes were selected. Next, PANTHER analysis facilitated the functional mapping of all 29 differentially expressed miRNAs to select 19 miRNAs related to metabolic processes. Finally, representative miRNAs and mRNAs were validated using qPCR and clinical characteristics. Integrating these results and interaction structure analysis, two miRNAs associated with SLE were selected. **b** Biological processes of the target genes of both differentially expressed and negatively regulated miRNAs. **c** Expression of candidate miRNAs in PBMCs of SLE patients and healthy controls. qRT-PCR was conducted on RNA samples from 25 SLE patients and 25 HCs. Data are presented as 2^−ΔCt^ relative to *U6* expression

Accordingly, a three-step analysis was conducted. First, by integrating the RNA-seq data, the small RNA-seq data, and the prediction of miRNA-target mRNA interactions across the six samples, 29 differentially expressed miRNAs were identified, including 10 that were downregulated and 19 that were upregulated in SLE, that showed a significant negative correlation with the expression of the target mRNA (Fig. 3a and Table 1). Next, considering that miRNAs play an important role in metabolic processes involved in SLE occurrence and development, PANTHER enrichment analysis was used to further select, among the 29 potential miRNAs, 19 candidate differentially expressed miRNAs that were associated with metabolic process: miR-1273h-5p, miR-874-5p, miR-330-5p, miR-342-5p, miR-361-5p, miR-4470, miR-6716-5p, miR-7854-3p, miR-92b-5p, NovelmiRNA-25, NovelmiRNA-296, NovelmiRNA-30, NovelmiRNA-376, NovelmiRNA-426, NovelmiRNA-489, NovelmiRNA-72, NovelmiRNA-815, NovelmiRNA-862, and NovelmiRNA-92 (Fig. 3b and Additional file 3: Table S7). Cytoscape software was used to construct the complex regulatory network of the 29 candidate miRNAs. As shown in Additional file 1A, miRNAs and target genes interacted in the miRNA-target mRNA regulatory network. Third, after applying the criteria of binding energy < − 31 kcal/mol, strict matching of the 2–8 bp of the miRNA seed region with the 3′UTR region of the target gene, and miRNA target gene expression level of > |2.9| (log_2_foldchange), three miRNAs that were upregulated (NovelmiRNA-25, all_hsa-miR-92b-5p and miR-1273h-5p) and one miRNA that was downregulated (miR-874-5p) in SLE patients were selected. The expression of three candidate miRNAs of them was validated in the validation cohort using qRT-PCR. All three miRNAs showed similar expression patterns between the qRT-PCR and NGS analyses. However, only NovelmiRNA-25 (P = 0.018) and miR-1273h-5p (P = 0.001) showed significant differential expression between the PBMCs of patients with SLE and those OF healthy controls (Fig. 3c). Therefore, these two miRNAs were considered candidates for further analysis.

### Correlation between candidate miRNA expression and SLE clinical characteristics

**Fig. 4:**
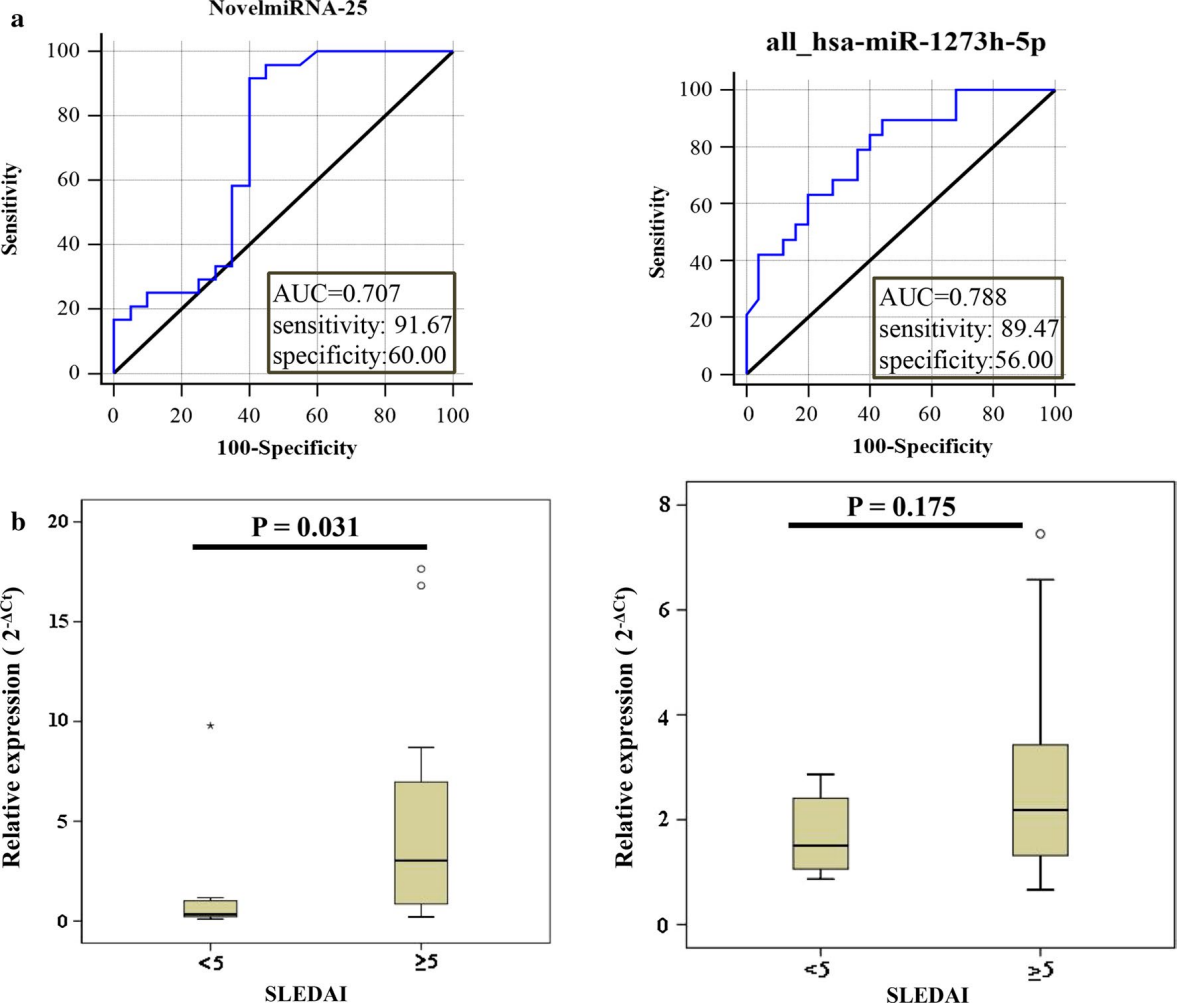
Correlation between expression of selected miRNAs and SLE clinical characteristics. **a** ROC curve analysis of NovelmiRNA-25 and miR-1273h-5p expression in patients with SLE. **b** Expression of NovelmiRNA-25 and miR-1273h-5p in active group and stable group

Using ROC curve analysis, the potential utility of NovelmiRNA-25 and miR-1273h-5p as new diagnostic biomarkers of SLE was explored further. As shown in Fig. 4a, the AUCs for NovelmiRNA-25 and miR-1273h-5p when distinguishing SLE patients from healthy controls were 0.707 and 0.788, respectively. The diagnostic sensitivities of NovelmiRNA-25 and miR-1273h-5p were 0.91 and 0.89, respectively, and the specificities were 0.60 and 0.56, respectively.

The relationship between candidate miRNA expression levels in PBMCs and the clinical characteristic of patients with SLE was investigated (Table 2). The expression of NovelmiRNA-25 in SLE patients was significantly correlated with leukocyte count (P = 0.045), neutrophil count (P = 0.002), albumin levels (P = 0.034), and low C3 (P = 0.019). Moreover, NovelmiRNA-25 expression in PBMCs was significantly associated with disease activity in patients with SLE, in both the active and stable groups (P < 0.05) (Fig. 4b).

**Table 2:** Correlation of the expression of the two candidate miRNA and clinical features of SLE

Clinical characteristics	N	NovelmiRNA-25	P	all-hsa-miR-1273h-5p	P
Leukocyte					
< 3.5	2	0.39 ± 0.12	0.045	1.50 ± 0.67	0.452
3.5–9.5	16	5.80 ± 5.38		2.78 ± 2.00	
> 9.5	3	0.64 ± 0.25		1.89 ± 1.02	
Neutrophil					
1.8–6.3	15	6.06 ± 5.48	0.002	2.91 ± 2.01	0.271
> 6.3	4	0.78 ± 0.34		1.72 ± 0.90	
Albumin					
< 40	14	4.89 ± 4.85	0.034	2.98 ± 1.98	0.748
40–55	1	16.81		3.65	
C3					
< 0.79	15	5.67 ± 5.74	0.019	2.86 ± 2.04	0.173
0.79–1.52	7	1.55 ± 1.61		1.73 ± 0.72	
C4					
< 0.16	17	4.68 ± 5.76	0.524	1.75 (1.15, 2.57)	0.031
0.16–0.38	4	2.74 ± 2.12		3.91 (2.45, 6.17)	
Lupus anticoagulant					
0.75–1.25	10	3.65 ± 5.57	0.561	2.11 ± 1.24	0.034
> 1.25	3	5.62 ± 4.57		4.58 ± 2.50	
24 h urine protein					
< 0.5	3	8.65 ± 8.17	0.074	4.36 ± 2.80	0.073
> 0.5	9	2.80 ± 2.73		2.11 ± 1.25	
Alanine aminotransferase					
7–40	14	5.36 ± 5.96	0.984	2.79 ± 1.98	0.61
> 40	2	5.45 ± 0.03		3.57 ± 1.96	
Anti-ds-DNA antibody					
Positive	12	1.99 (0.53, 6.59)	0.75	1.64 (1.07, 4.62)	0.553
Negative	6	2.10 (0.73, 11.75)		1.64 (1.07, 4.62)	

*C3/C4* complement 3/complement 4

Similarly, low expression of hsa-miR-1273h-5p in SLE patients was correlated with low C4 (P = 0.031) and lupus anticoagulant (P = 0.034). However, no significant association between hsa-miR-1273h-5p expression in PBMCs and disease activity in patients with SLE was observed, in either the active or the stable groups (P = 0.175) (Fig. 4b).

### NovelmiRNA-25 directly targets AMPD2

**Fig. 5:**
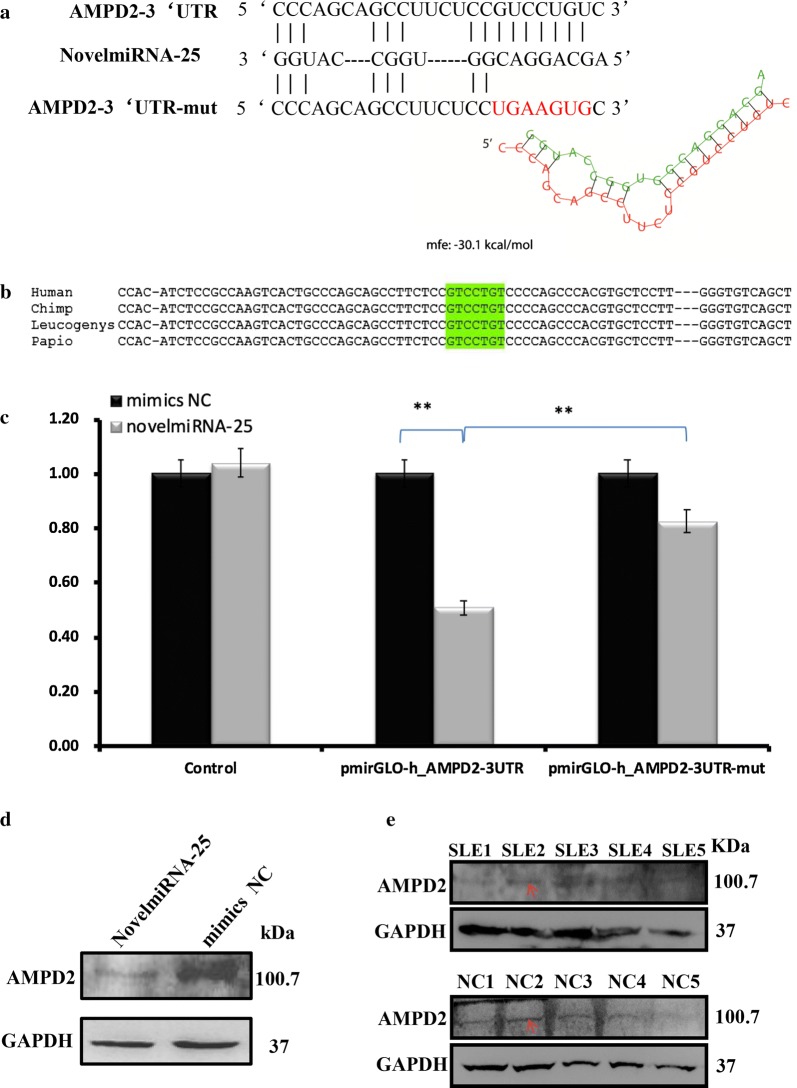
NovelmiRNA-25 targets *AMPD2*. **a** Predicted target sites in the *AMPD2* 3′UTR based on the NovelmiRNA-25 seed region. Mutations in the *AMPD2* 3′UTR generated missense sequences unable to pair with the NovelmiRNA-25 seed region. The structure of NovelmiRNA-25 bound to *AMPD2* is depicted. **b** NovelmiRNA-25 and the *AMPD2* 3′UTR seeding region, highlighted in green, are highly conserved in mammals. **c** Activity of the luciferase gene linked to the 3′UTR of *AMPD2*. pmirGLO firefly luciferase reporter plasmids with wild-type or mutated 3′UTR sequences of *AMPD2* were transiently transfected into cells with NovelmiRNA-25 precursor or negative control and a *Renilla* luciferase reporter for normalization. Luciferase activities were measured after 48 h. The mean of the results from the cells transfected with pmirGLO control vector was set as 100%. Data are the mean and standard deviation (SD) of separate transfections (n = 3). **d** Downregulation of endogenous AMPD2 protein expression by NovelmiRNA-25. Western blotting of AMPD2 protein after transfection with negative control (NC) or NovelmiRNA-25 mimic in HEK293T cells. Expression levels were normalized to that of GAPDH; **P < 0.05; AMDP2, adenosine monophosphate deaminase 2; **e** AMPD2 protein expression in PBMCs from SLE patients and negative control. Western blotting of AMPD2 protein after transfection with PBMCs. Expression levels were normalized to those of GAPDH. The arrow indicates the expression of AMPD2 protein

Sequencing analysis showed that NovelmiRNA-25 is located on chromosome 16:72089401–72089448. Next, the potential molecular targets of NovelmiRNA-25 were predicted. The coexpression network of NovelmiRNA-25 revealed an association with AMPD2 (Fig. 5a). The binding energy of NovelmiRNA-25 with *AMPD2* mRNA was − 30.1 kcal/mol. AMPD2 is involved in metabolic processes, including purine nucleobase metabolism (GO:0006144) and purine nucleotide metabolism (GO:0006163) (Additional file 3: Table S5). Moreover, the Thomson Reuters database showed that AMPD2 participates in various GO cellular processes, including cellular developmental process and cell differentiation (Additional file 2A and Additional file 3: Table S5). AMPD2 also plays a key role in the second-scored networks, including cell adhesion (42.0%), biological adhesion (42.0%), and the integrin–mediated signaling pathway (20.0%) (Additional file 2B and Additional file 3: Table S6). Therefore, we further explored the binding of NovelmiRNA-25 to the *AMPD2* 3′UTR. The sequences of both NovelmiRNA-25 and the *AMPD2* 3′UTR seeding region are highly conserved among primates and mammals (Fig. 5b).

To confirm further whether *AMPD2* is a direct downstream target of NovelmiRNA-25, a dual-luciferase reporter assay was performed, in which the direct binding of NovelmiRNA-25 to the pmirGLO *AMPD2* 3′UTR transcript repressed luciferase activity. HEK293T cells, which do not express NovelmiRNA-25, were used, and the expression of barely detectable levels of NovelmiRNA-25 was confirmed (data not shown). We observed that cotransfection of the NovelmiRNA-25 mimic suppressed the luciferase activity of pmirGLO with the *AMPD2* 3′UTR by 49% (P < 0.01); moreover, mutation of the NovelmiRNA-25-binding region within the *AMPD2* 3′UTR abrogated the reduction in the luciferase activity of the vector by 32% (P < 0.01) (Fig. 5c), indicating specificity of the target site of *AMPD2*.

Next, western blotting was performed to determine whether NovelmiRNA-25 downregulates the protein expression of AMPD2. At 48 h post-transfection with the Novel-miRNA-25 mimic, AMPD2 protein was significantly decreased in HEK293T cells transfected with Novel-miRNA-25 mimic compared with levels in cells transfected with the internal control (P < 0.05, Fig. 5d), and the expression of AMPD2 protein was significantly decreased in PBMCs of SLE patients compared with that in PBMCs from HCs (Fig. 5e). This finding indicates that overexpression of NovelmiRNA-25 regulates AMPD2 expression at the protein level, further confirming that NovelmiRNA-25 directly targets AMPD2.

## Discussion

In the current study, we conducted a multi-tiered integrative analysis using miRNA and mRNA sequencing in PBMCs from subjects with SLE and HCs for the first time. We identified 29 differentially expressed miRNAs, of which 19 were upregulated and 10 downregulated in SLE, with detectable negative regulatory effects on the expression of their target genes in PBMCs from SLE patients. Unlike in previous individual microarray studies [26–32], we performed NGS analysis of miRNAs and mRNAs from PBMCs of SLE patients simultaneously. Consequently, we identified new differentially expressed miRNAs that were not previously reported in SLE patients.

Metabolomic studies have identified the potential contributions of certain metabolites, including lipids, amino acids, nucleic acids, and carbohydrates, to inflammation; these, therefore, represent promising targets for the diagnosis and disease monitoring of SLE [27–29]. Therefore, we selected miRNAs involved in metabolic processes to explore potential links between metabolism and SLE. Among the three selected miRNAs associated with metabolic processes, NovelmiRNA-25 and miR-1273h-5p, which were upregulated in SLE, were validated by qRT-PCR in the validation cohort.

miRNAs may be differentially expressed in the PBMCs of SLE patients and healthy individuals. However, various studies have reported conflicting data (studies are listed in Additional file 3: Table S8). Previous studies have found that miR-19b, miR-20a [30], miR-126 [31], miR-125b [32], miR-27a [33], miR-155, miR-181b, miR-17 [34], miR-7 [35], and miR-210 [36] are dysregulated in the PBMCs of SLE patients resulting in the aberrant expression of essential target genes related to the pathogenesis and development of SLE, such as PTEN [35] and HIF-1α [36]. In contrast to these previous studies, we identified two novel miRNAs, NovelmiRNA-25 and miR-1273h-5p, that may be involved in the development of SLE.

According to the known miRNA targets published in several databases, we found that the metabolism-associated target of NovelmiRNA-25 was AMPD2, which is involved in purine nucleotide catabolism [37, 38]. The miRNA-target gene interaction results revealed that AMPD2 participates in the top-scored networks, including essential biological and metabolomic processes. Among metabolic processes, AMPD2, involved in purine biosynthesis and metabolism, is essential for cellular energy homeostasis and nucleic acids synthesis [39]. Based on the GeneCard database [40], we found that AMPD2 was specifically expressed in PBMCs, including CD8^+^ T cells, CD4^+^ T cells, B cells, and monocytes. Notably, these clusters of cells participate in the pathogenesis of SLE [41, 42]. AMPD2, which is mainly produced in the liver and spleen [37], is an enzyme that converts AMP to IMP. As adenosine and ATP are both synthesized from AMP, AMPD2 was involved in several different metabolic pathways by mediating changes in adenosine and ATP levels. Downregulated AMPD2 contributed to AMP accumulation, which mediated higher adenosine and ATP levels. On the one hand, adenosine is known to possess anti-inflammatory and analgesic activities [43]. Koizumi et al. [44] reported that tofacitinib may increase the levels of adenosine in chondrocytes through the downregulation of AMPD2, indicating its anti-inflammatory activity. On the other hand, ATP is known to have pro-inflammatory effects and activates the inflammasome [45]. In patients with RA, high concentrations of ATP were found in the synovial fluid [46], implying that ATP was highly involved in RA. Moreover, some studies have revealed the pathologic roles of increased ATP in the development of inflammatory intestinal disorders [47]. Activated SLE increases the level of ATP, which possess pro-inflammation and immune affect, by regulating AMPD2 expression. Therefore, our results indicated that AMPD2 in SLE was more likely to be involved in the pro-inflammatory pathway by increasing ATP level. As AMPD2 is a metabolism-associated target gene of NovelmiRNA-25, these results indicate that NovelmiRNA-25 is a potential functional target in SLE.

Although upregulated miRNA miR-1273h-5p did not appear to be involved in disease flare-ups or organ injury in SLE, our findings did implicate miR-1273h-5p in the pathogenesis of SLE. miR-1273h-5p, with a length of 21 nt, is encoded by the intergenic sequence on chromosome 16 [48]. miR-1273h-5p was predicted to target five genes associated with metabolic processes; these genes encoded a peroxidase, glycosyltransferase, ATP synthase and hydrolase, carbohydrate phosphatase, and exoribonuclease. Additionally, miR-1273h-5p has been found to be dysregulated in cancers and other autoimmune diseases. For example, the expression of miR-1273 was shown to be increased in the pancreas of a mouse model of pancreatic cancer [49].

Numerous clinical manifestations of SLE are similar to those of other illnesses, making the disease difficult to diagnose or distinguish from other autoimmune diseases. Furthermore, there is no single laboratory indication that can be used for a definitive diagnosis of SLE [50]. In the present study, we found that miRNAs may be promising biomarkers for SLE diagnosis. An ROC curve was used to evaluate the expression of the two candidate miRNAs in the PBMCs of SLE patients, demonstrating that these miRNAs may possess clinical diagnostic value for distinguishing SLE patients from healthy individuals. Moreover, we explored the associations between these miRNAs and the clinical characteristics of SLE patients, including autoantibodies, complement, and other relevant features. The results showed that the expression of the two miRNAs was correlated with complement levels. Further analysis of miRNA levels between patients with active and stable SLE showed that the expression of NovelmiRNA-25 was positively correlated with the degree of SLE activity. This positive correlation suggests that NovelmiRNA-25 may be involved in progression and flare-ups as well as organ damage during SLE and indicates that it is a potential functional target in SLE. The results of a dual-luciferase reporter assay revealed that the overexpression of NovelmiRNA-25 resulted in the downregulation of *AMPD2* gene expression in PBMCs through direct targeting of the *AMPD2* 3′UTR. In addition, the results of western blotting showed that the overexpression of NovelmiRNA-25 downregulated endogenous AMPD2 protein levels in a human cell line. Indicating the function of NovelmiRNA-25 in regulating AMPD2. Moreover, AMPD2 expression in the active SLE group was lower than that in the stable SLE group (Additional file 1B), and AMPD2 protein levels in SLE patients were decreased compared to those in HCs, indicating that AMPD2 was associated with SLE. This evidence further supports the theory that NovelmiRNA-25 is a promising target in the development of therapeutic strategies for SLE.

## Conclusion

In summary, in the present work, miRNA and mRNA expression profiling in PBMCs from SLE patients and HCs identified miRNAs that are dysregulated in PBMCs from SLE patients. We further analyzed the regulatory networks of differentially expressed miRNAs and mRNAs and identified NovelmiRNA-25 and miR-1273h-5p as differentially expressed in SLE. The identification of these miRNAs may provide useful insights into the pathophysiology of SLE. Further experimental results point to a novel mechanism underlying SLE pathogenesis involving NovelmiRNA-25 and demonstrated the upregulation of this miRNA in SLE. Our study suggests that miRNAs represent novel diagnostic biomarkers, disease activity markers, and potential therapeutic targets for SLE. Further investigation is needed to clarify the roles of the identified miRNAs in the pathogenesis of SLE, with the aim of developing new methods for the diagnosis and treatment of SLE.

## Additional files

**Additional file 1.** A. Regulation network between miRNAs and mRNAs. The regulation network was drawn using Cytoscape. In the network, arrows represent miRNAs, and circles represent mRNAs. Yellow indicates upregulation, and blue indicates downregulation. B. Expression of AMPD2 in PBMCs of SLE patients. QRT-PCR was conducted on RNA samples from the active SLE group and stable SLE group among 25 SLE patients. Data are presented as 2^−ΔCt^ relative to 18S ribosomal RNA expression.

**Additional file 2.** Gene ontology (GO) and analyze network (AN) analyses of miRNA target genes in SLE. A. Gene ontology (GO) cellular processes. Sorting is according to “statistically significant processes”. B. AN top-scored pathways (by the number of pathways). Thick cyan lines indicate fragments of canonical pathways. Upregulated and downregulated genes are indicated by red and blue circles, respectively. The “checkerboard” pattern indicates mixed expression.

**Additional file 3: Table S1.** Clinical characteristics of SLE patients and healthy controls. **Table S2.** Primers for validation of miRNA and mRNA by quantitative PCR. **Table S3.** Clinical characteristics of patients under survey and the mapping properties of their RNA-seq and small RNA-seq datasets. **Table S4.** Differentially expressed miRNA in PBMCs of SLE patients and normal controls. **Table S5.** Enrichment by Gene Ontology cellular processes. **Table S6.** Most relevant networks of the target gene of miRNAs. **Table S7.** Target gene associated with the metabolic process. **Table S8.** miRNAs differentially involved in the development and function of SLE.

## Abbreviations

SLE: systemic lupus erythematosus
miRNAs: microRNAs
PBMCs: peripheral blood mononuclear cells
HCs: healthy controls
NSAIDs: non-steroidal anti-inflammatory drugs
NGS: next-generation sequencing
ACR: American College of Rheumatology
SLEDAI: Systemic Lupus Erythematosus Disease Activity Index
GO: gene ontology
KEGG: Kyoto Encyclopedia of Genes and Genome
DEGs: differentially expressed genes
BP: biological process
CC: cellular component
MF: molecular function
ROC: receiver operating characteristic
AUC: areas under curve
