# Supplementary figures and images for: NovelmiRNA-25 inhibits AMPD2 in peripheral blood mononuclear cells of patients with systemic lupus erythematosus and represents a promising novel biomarker
Source: J Transl Med. 2018 Dec 22;16:370. doi: 10.1186/s12967-018-1739-5 (PMC6303892; doi:10.1186/s12967-018-1739-5)

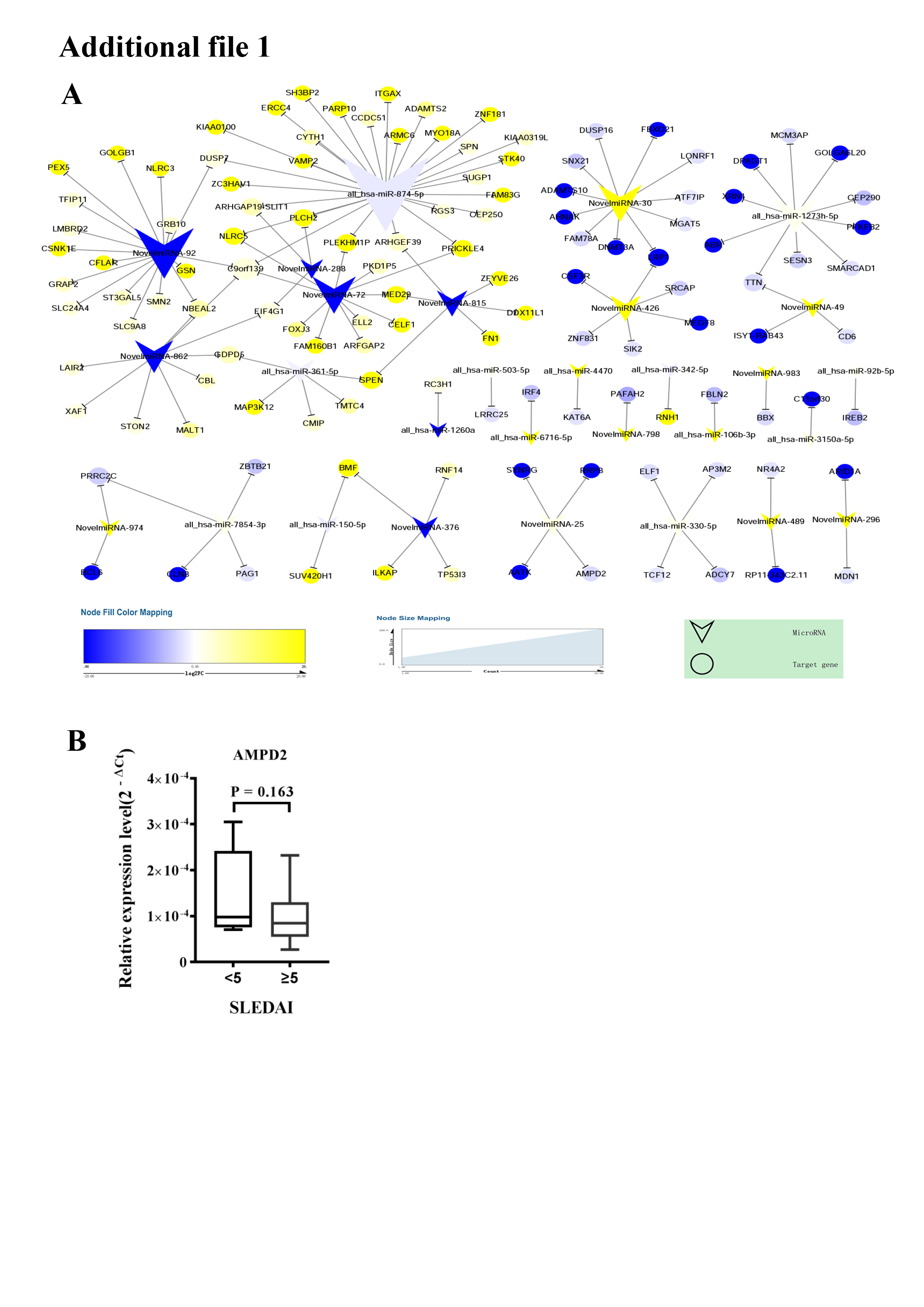

Supplement: Supplementary file 1 — Additional file 1. A. Regulation network between miRNAs and mRNAs. The regulation network was drawn using Cytoscape. In the network, arrows represent miRNAs, and circles represent mRNAs. Yellow indicates upregulation, and blue indicates downregulation. B. Expression of AMPD2 in PBMCs of SLE patients. QRT-PCR was conducted on RNA samples from the active SLE group and stable SLE group among 25 SLE patients. Data are presented as 2−ΔCt relative to 18S ribosomal RNA expression. [file 12967_2018_1739_MOESM1_ESM.tif]

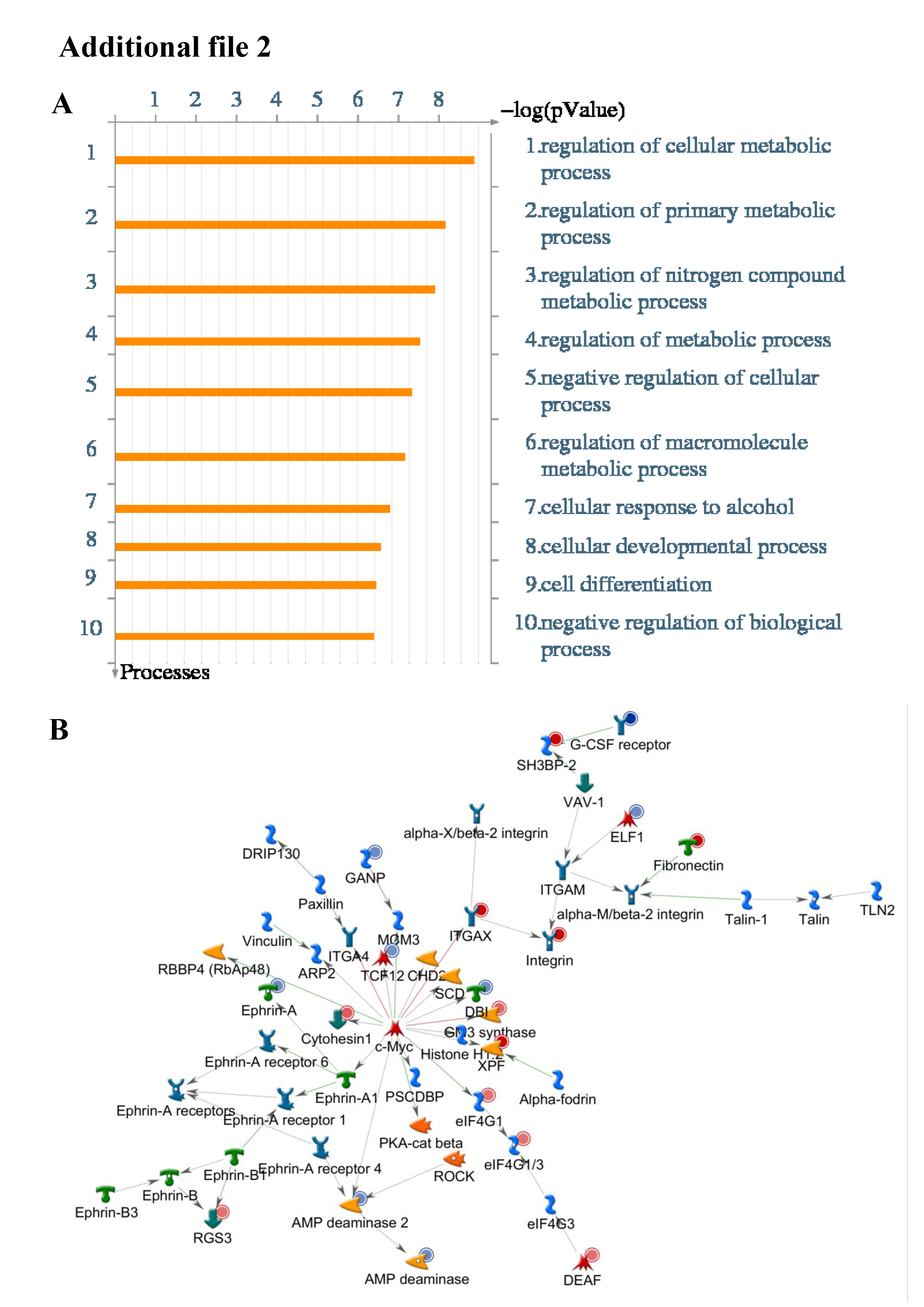

Supplement: Supplementary file 2 — Additional file 2. Gene ontology (GO) and analyze network (AN) analyses of miRNA target genes in SLE. A. Gene ontology (GO) cellular processes. Sorting is according to “statistically significant processes”. B. AN top-scored pathways (by the number of pathways). Thick cyan lines indicate fragments of canonical pathways. Upregulated and downregulated genes are indicated by red and blue circles, respectively. The “checkerboard” pattern indicates mixed expression. [file 12967_2018_1739_MOESM2_ESM.tif]
