# Supplementary material for: NovelmiRNA-25 inhibits AMPD2 in peripheral blood mononuclear cells of patients with systemic lupus erythematosus and represents a promising novel biomarker
Source: J Transl Med. 2018 Dec 22;16:370. doi: 10.1186/s12967-018-1739-5 (PMC6303892; doi:10.1186/s12967-018-1739-5)
Supplement: Supplementary file 3 — Additional file 3: Table S1. Clinical characteristics of SLE patients and healthy controls. Table S2. Primers for validation of miRNA and mRNA by quantitative PCR. Table S3. Clinical characteristics of patients under survey and the mapping properties of their RNA-seq and small RNA-seq datasets. Table S4. Differentially expressed miRNA in PBMCs of SLE patients and normal controls. Table S5. Enrichment by Gene Ontology cellular processes. Table S6. Most relevant networks of the target gene of miRNAs. Table S7. Target gene associated with the metabolic process. Table S8. miRNAs differentially involved in the development and function of SLE. [file 12967_2018_1739_MOESM3_ESM.docx]

**Additional file 3:**

**Table S1. Clinical characteristics of SLE patients and healthy controls.**

| Clinical characteristics^#^ | Exploratory cohort (n=6) | | Validation cohort (n = 50) | |
| --- | --- | --- | --- | --- |
|  | SLE (n=3) | Healthy control (n=3) | SLE (n = 25) | Healthy control (n=25) |
| Sex, male/female | 0/3 | 0/3 | 2/23 | 4/21 |
| Age (year) | 43.0±8.54 | 27.67±3.51 | 28.63±10.25 | 27.98±10.89 |
| SLEDAI scores | 15.33±1.54 |  | 9.09±5.79 |  |
| ANA | 3(3) |  | 16(19) |  |
| Anti-ds-DNA antibody | 3(3) |  | 9(19) |  |
| Lupus anticoagulant | 1.45±0.48 |  | 0.95±0.35 |  |
| leukocyte | 6.02±1.37 |  | 6.21±2.70 |  |
| Neutrophils | 4.49±0.38 |  | 4.40±2.25 |  |
| Hemoglobin | 89.67±13.05 |  | 103.0±26.24 |  |
| Platelets | 206.0±9.64 |  | 183.83±77.10 |  |
| ALT |  |  | 21.13±13.66 |  |
| Low C3 level | 2(2) |  | 15(22) |  |
| Low C4 level | 2(2) |  | 17(22) |  |
| Low albumin level |  |  | 11(14) |  |
| Urine protein positive | 2(2) |  | 15(21) |  |

^#^C3/C4 complement 3/complement 4; SLEDAI, systemic lupus erythematosus disease activity index; ALT, alanine aminotransferase

**Table S2. Primers for validation of miRNA and mRNA by quantitative PCR.**

|  | **Primer** | **Sequence** | **TM (℃)** |
| --- | --- | --- | --- |
| hsa-miR-874-5p | RT stem-loop primer | GTCGTATCCAGTGCAGGGTCCGAGGTATTCGCACTGGATACGACAGTCGG | 58 |
|  | Forward primer | TAACTGCCCTGGCCCGAG |  |
| hsa-miR-1273h-5p | RT stem-loop primer | GTCGTATCCAGTGCAGGGTCCGAGGTATTCGCACTGGATACGACGCCTGG | 58 |
|  | Forward primer | TTCTGCAGACTCGACCTCCC |  |
| NovelmiRNA-25 | RT stem-loop primer | GTCGTATCCAGTGCAGGGTCCGAGGTATTCGCACTGGATACGACCCATGG | 58 |
|  | Forward primer | AAACAGCAGGACGGTGGC |  |
| Common reverse primer | | CAGTGCAGGGTCCGAGGTAT |  |
| U6 | Forward primer | CTCGCTTCGGCAGCACATATACT | 58 |
|  | Reverse prime | ACGCTTCACGAATTTGCGTGTC |  |
| AMPD2 | Forward primer | ATGCCAAATACAACCCTAT | 57.3 |
|  | Reverse prime | ACCTCCTTGATGATGTGA |  |
| 18S ribosomal RNA | Forward primer | ACAGGATTGACAGATTGA | 58 |
|  | Reverse prime | TATCGGAATTAACCAGACA |  |

**Table S3. Clinical characteristics of patients under survey and the mapping properties of their RNA-seq and small RNA-seq datasets.**

| Clinical characteristics | | | | | RNA-Seq | | | Small RNA | | |
| --- | --- | --- | --- | --- | --- | --- | --- | --- | --- | --- |
| Case No. | Sex | Age | Type | SLEDAI | Total Reads (Millions) | Uniquely Mapped Reads (Millions) | Detected Genes (Thousands)^*^ | Total Reads (Thousands) | Reads mapped to miRNAs (Thousands) | Detected miRNAs (Thousands)^**^ |
| 1 | F | 35 | SLE | 16 | 92.6 | 80.9 | 27.3 | 307.0 | 150.3 | 570.8 |
| 2 | F | 52 | SLE | 16 | 100.6 | 91.9 | 26.8 | 297.5 | 142.8 | 1032.2 |
| 3 | F | 42 | SLE | 14 | 94.7 | 86.3 | 26.3 | 290.1 | 134.4 | 466.8 |
| 4 | F | 31 | N | ／ | 117.5 | 109.7 | 27.3 | 350.6 | 179.6 | 656.9 |
| 5 | F | 24 | N | ／ | 105.2 | 98.2 | 26.7 | 312.8 | 151.5 | 664.2 |
| 6 | F | 28 | N | ／ | 105.8 | 98.4 | 26.9 | 321.8 | 157.7 | 715.1 |

* RPKM > 0.05; ** with mapped read.

**Table S4. Differentially expressed miRNA in PBMCs of SLE patients and normal controls.**

| miRNA | Length | log2^FoldChange^ | P Value | Regulation |
| --- | --- | --- | --- | --- |
| all_hsa-miR-1260a | 18 | NA**^#^** | 7.69E-07 | Down |
| NovelmiRNA-749 | 21 | NA | 3.80E-06 | Down |
| NovelmiRNA-72 | 23 | NA | 4.65E-06 | Down |
| NovelmiRNA-822 | 22 | Inf | 6.19E-05 | Up |
| NovelmiRNA-641 | 18 | Inf**^*^** | 7.59E-05 | Up |
| all_hsa-miR-5010-5p | 23 | Inf | 0.0001 | Up |
| all_hsa-miR-31-5p | 23 | -2.523937415 | 0.0003 | Down |
| all_hsa-miR-100-5p | 22 | -1.853323671 | 0.0004 | Down |
| all_hsa-miR-4470 | 23 | Inf | 0.0007 | Up |
| NovelmiRNA-999 | 23 | NA | 0.0010 | Down |
| NovelmiRNA-798 | 21 | Inf | 0.0011 | Up |
| all_hsa-miR-142-5p | 22 | -2.182228317 | 0.0015 | Down |
| all_hsa-miR-150-5p | 22 | -1.522629906 | 0.0029 | Down |
| NovelmiRNA-376 | 25 | NA | 0.0032 | Down |
| all_hsa-miR-139-5p | 23 | 1.51768942 | 0.0040 | Up |
| all_hsa-miR-618 | 23 | -2.095089279 | 0.0052 | Down |
| all_hsa-miR-873-5p | 18 | -2.724269632 | 0.0063 | Down |
| all_hsa-miR-330-5p | 25 | 1.468765481 | 0.0068 | Up |
| all_hsa-miR-29b-2-5p | 20 | -1.385815083 | 0.0103 | Down |
| all_hsa-miR-29b-1-5p | 20 | -1.385093519 | 0.0104 | Down |
| all_hsa-miR-708-5p | 21 | -6.334549999 | 0.0109 | Down |
| NovelmiRNA-713 | 22 | Inf | 0.0111 | Up |
| all_hsa-miR-29a-5p | 22 | -1.323874238 | 0.0111 | Down |
| all_hsa-miR-874-5p | 24 | -1.713221694 | 0.0114 | Down |
| all_hsa-miR-342-5p | 24 | -1.30266414 | 0.0122 | Down |
| all_hsa-miR-106b-3p | 20 | Inf | 0.0128 | Up |
| NovelmiRNA-773 | 23 | Inf | 0.0128 | Up |
| all_hsa-miR-1260b | 18 | -1.241840636 | 0.0136 | Down |
| NovelmiRNA-815 | 20 | NA | 0.0149 | Down |
| NovelmiRNA-288 | 18 | NA | 0.0150 | Down |
| NovelmiRNA-573 | 23 | NA | 0.0189 | Down |
| NovelmiRNA-319 | 23 | Inf | 0.0209 | Up |
| NovelmiRNA-358 | 20 | -1.400712165 | 0.0210 | Down |
| all_hsa-miR-7854-3p | 23 | 1.704315921 | 0.0214 | Up |
| NovelmiRNA-956 | 22 | NA | 0.0224 | Down |
| NovelmiRNA-360 | 19 | NA | 0.0233 | Down |
| NovelmiRNA-983 | 24 | Inf | 0.0237 | Up |
| NovelmiRNA-489 | 22 | Inf | 0.0239 | Up |
| all_hsa-miR-200a-5p | 21 | -1.554775957 | 0.0240 | Down |
| NovelmiRNA-273 | 23 | -3.81756952 | 0.0243 | Down |
| NovelmiRNA-862 | 23 | NA | 0.0244 | Down |
| NovelmiRNA-144 | 22 | Inf | 0.0247 | Up |
| NovelmiRNA-30 | 21 | Inf | 0.0282 | Up |
| all_hsa-miR-3150a-5p | 22 | 2.296785856 | 0.0315 | Up |
| all_hsa-miR-101-3p | 21 | -1.158517051 | 0.0322 | Down |
| all_hsa-miR-101-5p | 21 | -1.153038733 | 0.0331 | Down |
| all_hsa-miR-361-5p | 22 | -1.103414368 | 0.0337 | Down |
| all_hsa-miR-3064-5p | 22 | Inf | 0.0340 | Up |
| NovelmiRNA-410 | 22 | NA | 0.0343 | Down |
| NovelmiRNA-426 | 24 | Inf | 0.0344 | Up |
| NovelmiRNA-209 | 22 | Inf | 0.0345 | Up |
| all_hsa-miR-1273h-5p | 22 | 1.169180254 | 0.0376 | Up |
| all_hsa-miR-92b-5p | 22 | 1.212699002 | 0.0393 | Up |
| NovelmiRNA-92 | 22 | NA | 0.0396 | Down |
| all_hsa-miR-503-5p | 21 | 1.245779356 | 0.0409 | Up |
| all_hsa-miR-148a-3p | 22 | 1.051637134 | 0.0417 | Up |
| all_hsa-miR-148a-5p | 22 | 1.051637134 | 0.0417 | Up |
| all_hsa-miR-3120-5p | 20 | 1.466721814 | 0.0421 | Up |
| all_hsa-miR-128-1-5p | 23 | 1.342335156 | 0.0429 | Up |
| all_hsa-miR-6716-5p | 24 | Inf | 0.0433 | Up |
| NovelmiRNA-296 | 21 | Inf | 0.0433 | Up |
| NovelmiRNA-49 | 22 | Inf | 0.0447 | Up |
| NovelmiRNA-299 | 23 | 3.423170074 | 0.0476 | Up |
| NovelmiRNA-25 | 19 | 2.336817328 | 0.0484 | Up |
| NovelmiRNA-974 | 22 | Inf | 0.0488 | Up |
| all_hsa-miR-625-5p | 23 | 2.143069035 | 0.0499 | Up |

NA^#^: Only expressed in Normal controls; Inf *: Only expressed in SLE patients.

**Table S5. Enrichment by Gene Ontology cellular processes.**

|  | Processes | Total | pValue | Min FDR | p-value | FDR | In Data | Network Objects from Active Data |
| --- | --- | --- | --- | --- | --- | --- | --- | --- |
| 1 | regulation of cellular metabolic process | 8223 | 1.47E-09 | 4.7749E-06 | 1.465E-09 | 4.77E-06 | 80 | GOLGB1, ZPK(MAP3K12), Casein kinase I epsilon, Casein kinase I, FOXJ3, CLN8, Spectrin, SPTBN(spectrin1-4), BBX, F262, MED29, ELOF1, Fibronectin, RBM15/MKL1 fusion protein, RBM15, MKP-7, XPF, TCF12, DNMT3A, TFIP11, LONRF1, mGluR7, Galpha(i)-specific metabotropic glutamate GPCRs, Roquin, KIAA0409, CUGBP1, BAF250, BAF250A, ZNF295, Cep290, MOZ, IRP, IRP2, FAM83G, Bcl-6, EMSY, NURR1, Rnf14 (ARA54), Sestrin 3, eIF4G1/3, eIF4G1, SHARP (SPEN), NLRC5, ZC3HAV1, MKP-X, CD43, ZNF831, SUV420H1, c-Cbl, DEAF, STK40, ELF1, MALT1, GRB10, c-FLIP, Integrin, Gelsolin, ZNF181, BMF, Ephrin-A, Adenylate cyclase type VII, Adenylate cyclase, RGS3, C1D, ATF7IP, A2M receptor, Spinophilin, IRF4, CD6, DBI, SMARCAD1, PP2C-DELTA, SrCap, MEGF8, SMN, ELL2, NOD3, XRN1, PARP-10, Titin |
| 2 | regulation of primary metabolic process | 8148 | 7.36E-09 | 1.1991E-05 | 7.358E-09 | 1.2E-05 | 78 | GOLGB1, ZPK(MAP3K12), Casein kinase I epsilon, Casein kinase I, FOXJ3, CLN8, Spectrin, SPTBN(spectrin1-4), BBX, F262, MED29, ELOF1, Fibronectin, RBM15/MKL1 fusion protein, RBM15, MKP-7, XPF, TCF12, DNMT3A, TFIP11, LONRF1, mGluR7, Galpha(i)-specific metabotropic glutamate GPCRs, Roquin, KIAA0409, CUGBP1, BAF250, BAF250A, ZNF295, Cep290, MOZ, IRP, IRP2, FAM83G, Bcl-6, EMSY, NURR1, Rnf14 (ARA54), eIF4G1/3, eIF4G1, SHARP (SPEN), NLRC5, ZC3HAV1, MKP-X, CD43, ZNF831, SUV420H1, c-Cbl, DEAF, STK40, ELF1, MALT1, GRB10, c-FLIP, Integrin, Gelsolin, ZNF181, Ephrin-A, Adenylate cyclase type VII, Adenylate cyclase, RGS3, C1D, ATF7IP, A2M receptor, Spinophilin, IRF4, CD6, DBI, SMARCAD1, PP2C-DELTA, SrCap, MEGF8, SMN, ELL2, NOD3, XRN1, PARP-10, Titin |
| 3 | regulation of nitrogen compound metabolic process | 7918 | 1.35E-08 | 1.4625E-05 | 1.346E-08 | 1.46E-05 | 76 | GOLGB1, ZPK(MAP3K12), Casein kinase I epsilon, Casein kinase I, FOXJ3, CLN8, Spectrin, SPTBN(spectrin1-4), BBX, F262, MED29, ELOF1, Fibronectin, RBM15/MKL1 fusion protein, RBM15, MKP-7, XPF, TCF12, DNMT3A, TFIP11, LONRF1, mGluR7, Galpha(i)-specific metabotropic glutamate GPCRs, Roquin, KIAA0409, CUGBP1, BAF250, BAF250A, ZNF295, Cep290, MOZ, IRP, IRP2, FAM83G, Bcl-6, EMSY, NURR1, Rnf14 (ARA54), eIF4G1/3, eIF4G1, SHARP (SPEN), NLRC5, ZC3HAV1, MKP-X, CD43, ZNF831, SUV420H1, c-Cbl, DEAF, STK40, ELF1, MALT1, c-FLIP, Integrin, Gelsolin, ZNF181, Ephrin-A, Adenylate cyclase type VII, Adenylate cyclase, RGS3, C1D, ATF7IP, A2M receptor, Spinophilin, IRF4, CD6, SMARCAD1, PP2C-DELTA, SrCap, MEGF8, SMN, ELL2, NOD3, XRN1, PARP-10, Titin |
| 4 | regulation of metabolic process | 8898 | 3.22E-08 | 2.625E-05 | 3.222E-08 | 2.62E-05 | 81 | GOLGB1, ZPK(MAP3K12), Casein kinase I epsilon, Casein kinase I, FOXJ3, CLN8, Spectrin, SPTBN(spectrin1-4), BBX, F262, MED29, ELOF1, Fibronectin, RBM15/MKL1 fusion protein, RBM15, MKP-7, XPF, TCF12, DNMT3A, TFIP11, LONRF1, mGluR7, Galpha(i)-specific metabotropic glutamate GPCRs, Roquin, KIAA0409, CUGBP1, BAF250, BAF250A, ZNF295, Cep290, MOZ, IRP, IRP2, FAM83G, Bcl-6, EMSY, NURR1, Rnf14 (ARA54), Sestrin 3, eIF4G1/3, eIF4G1, SHARP (SPEN), NLRC5, ZC3HAV1, MKP-X, CD43, ZNF831, SUV420H1, c-Cbl, DEAF, STK40, ELF1, MALT1, GRB10, c-FLIP, Integrin, Gelsolin, ZNF181, BMF, Ephrin-A, Adenylate cyclase type VII, Adenylate cyclase, RGS3, C1D, ATF7IP, A2M receptor, Spinophilin, IRF4, CD6, DBI, SMARCAD1, PP2C-DELTA, SrCap, MEGF8, SMN, ELL2, NOD3, XRN1, PARP-10, Titin, RAI |
| 5 | negative regulation of cellular process | 6398 | 5.17E-08 | 3.3683E-05 | 5.168E-08 | 3.37E-05 | 65 | ZPK(MAP3K12), Casein kinase I epsilon, Casein kinase I, CLN8, Spectrin, Beta-fodrin, SPTBN(spectrin1-4), Fibronectin, RBM15/MKL1 fusion protein, RBM15, MKP-7, XPF, DNMT3A, PAFAH2, TFIP11, mGluR7, Galpha(i)-specific metabotropic glutamate GPCRs, Roquin, KIAA0409, CUGBP1, BAF250, BAF250A, ZNF295, MOZ, IRP, IRP2, Bcl-6, NURR1, ABR, Sestrin 3, eIF4G1/3, eIF4G1, SHARP (SPEN), NLRC5, PAG, MKP-X, CD43, ZNF831, PTS1 receptor, c-Cbl, DEAF, STK40, ELF1, MALT1, GRB10, c-FLIP, Integrin, Gelsolin, BMF, Myosin XVIIIA, Ephrin-A, Adenylate cyclase, RGS3, C1D, ATF7IP, GDPD5, A2M receptor, AATK, Spinophilin, IRF4, DBI, XAF1, NOD3, XRN1, PARP-10 |
| 6 | regulation of macromolecule metabolic process | 8216 | 7.90E-08 | 4.2926E-05 | 7.903E-08 | 4.29E-05 | 76 | GOLGB1, ZPK(MAP3K12), Casein kinase I epsilon, Casein kinase I, FOXJ3, CLN8, Spectrin, SPTBN(spectrin1-4), BBX, MED29, ELOF1, Fibronectin, RBM15/MKL1 fusion protein, RBM15, MKP-7, XPF, TCF12, DNMT3A, TFIP11, LONRF1, Galpha(i)-specific metabotropic glutamate GPCRs, Roquin, KIAA0409, CUGBP1, BAF250, BAF250A, ZNF295, Cep290, MOZ, IRP, IRP2, FAM83G, Bcl-6, EMSY, NURR1, Rnf14 (ARA54), eIF4G1/3, eIF4G1, SHARP (SPEN), NLRC5, ZC3HAV1, MKP-X, CD43, ZNF831, SUV420H1, c-Cbl, DEAF, STK40, ELF1, MALT1, GRB10, c-FLIP, Integrin, Gelsolin, ZNF181, Ephrin-A, Adenylate cyclase type VII, Adenylate cyclase, RGS3, C1D, ATF7IP, A2M receptor, Spinophilin, IRF4, CD6, SMARCAD1, PP2C-DELTA, SrCap, MEGF8, SMN, ELL2, NOD3, XRN1, PARP-10, Titin, RAI |
| 7 | cellular response to alcohol | 151 | 1.84E-07 | 8.587E-05 | 1.844E-07 | 8.59E-05 | 9 | Spectrin, SPTBN(spectrin1-4), Fibronectin, DNMT3A, IRP, IRP2, Adenylate cyclase type VII, Adenylate cyclase, XRN1 |
| 8 | cellular developmental process | 5333 | 3.07E-07 | 0.00012524 | 3.074E-07 | 0.000125 | 56 | Casein kinase I epsilon, Casein kinase I, FOXJ3, CLN8, Spectrin, Beta-fodrin, SPTBN(spectrin1-4), **AMP deaminase 2**, AMP deaminase, Fibronectin, RBM15/MKL1 fusion protein, TCF12, DNMT3A, TFIP11, Galpha(i)-specific metabotropic glutamate GPCRs, Roquin, CUGBP1, BAF250, BAF250A, Cep290, MOZ, IRP, IRP2, Bcl-6, NURR1, eIF4G1/3, eIF4G1, SHARP (SPEN), CD43, ARMC6, PTS1 receptor, DEAF, ELF1, XTP2, MALT1, GRB10, c-FLIP, NBEAL2, Integrin, Gelsolin, STN2, Ephrin-A, Ephrin-A2, PAPP-A, Adenylate cyclase, GDPD5, A2M receptor, AATK, Spinophilin, IRF4, GRAP2, DBI, MEGF8, SMN, XRN1, Titin |
| 9 | cell differentiation | 5228 | 4.00E-07 | 0.00014018 | 3.999E-07 | 0.00014 | 55 | Casein kinase I epsilon, Casein kinase I, FOXJ3, CLN8, Spectrin, Beta-fodrin, SPTBN(spectrin1-4), **AMP deaminase 2**, AMP deaminase, Fibronectin, RBM15/MKL1 fusion protein, TCF12, DNMT3A, TFIP11, Galpha(i)-specific metabotropic glutamate GPCRs, Roquin, CUGBP1, BAF250, BAF250A, Cep290, MOZ, IRP, IRP2, Bcl-6, NURR1, eIF4G1/3, eIF4G1, SHARP (SPEN), CD43, ARMC6, PTS1 receptor, DEAF, ELF1, XTP2, MALT1, GRB10, c-FLIP, NBEAL2, Integrin, Gelsolin, STN2, Ephrin-A, Ephrin-A2, PAPP-A, Adenylate cyclase, GDPD5, A2M receptor, AATK, Spinophilin, IRF4, GRAP2, DBI, MEGF8, SMN, Titin |
| 10 | negative regulation of biological process | 7068 | 4.54E-07 | 0.00014018 | 4.539E-07 | 0.00014 | 67 | ZPK(MAP3K12), Casein kinase I epsilon, Casein kinase I, CLN8, Spectrin, Beta-fodrin, SPTBN(spectrin1-4), Fibronectin, RBM15/MKL1 fusion protein, RBM15, MKP-7, XPF, DNMT3A, PAFAH2, TFIP11, mGluR7, Galpha(i)-specific metabotropic glutamate GPCRs, Roquin, KIAA0409, CUGBP1, BAF250, BAF250A, ZNF295, MOZ, IRP, IRP2, Bcl-6, NURR1, ABR, Sestrin 3, eIF4G1/3, eIF4G1, SHARP (SPEN), NLRC5, PAG, ZC3HAV1, MKP-X, CD43, ZNF831, PTS1 receptor, c-Cbl, DEAF, STK40, ELF1, MALT1, GRB10, c-FLIP, Integrin, Gelsolin, BMF, Myosin XVIIIA, Ephrin-A, Adenylate cyclase, RGS3, C1D, ATF7IP, GDPD5, A2M receptor, AATK, Spinophilin, IRF4, DBI, XAF1, NOD3, XRN1, PARP-10, RAI |

**Table S6. Most relevant networks of the target gene of miRNAs.**

| No | Network name | Processes | Size | Target | Pathways | p‑Value | zScore | gScore |
| --- | --- | --- | --- | --- | --- | --- | --- | --- |
| 1 | Ephrin‑A, Fibronectin, Ephrin‑A2, c‑Src, FAK1 | ephrin receptor signaling pathway (63.8%), transmembrane receptor protein tyrosine kinase signaling pathway (72.3%), enzyme linked receptor protein signaling pathway (78.7%), movement of cell or subcellular component (91.5%), cell morphogenesis (76.6%) | 52 | 3 | 105 | 6.84e‑06 | 15.69 | 146.94 |
| 2 | AMP deaminase 2, RGS3, SH3BP‑2, XPF, ELF1 | ephrin receptor signaling pathway (22.0%), cell adhesion (42.0%), biological adhesion (42.0%), integrin‑mediated signaling pathway (20.0%), cell‑substrate adhesion (22.0%) | 50 | 19 | 0 | 1.56e‑47 | 100.38 | 100.38 |
| 3 | XTP2, IRP2, BBX, FOXJ3, c‑Myc | activation of protein kinase A activity (16.0%), cAMP biosynthetic process (16.0%), cGMP biosynthetic process (16.0%), response to platelet aggregation inhibitor (16.0%), regulation of actin filament polymerization (24.0%) | 50 | 12 | 0 | 1.02e‑27 | 63.98 | 63.98 |

**Table S7. Target gene associated with the metabolic process.**

| Number | miRNA | Target gene | PANTHER Protein Class | 3'UTR Binding Energy (kcal/mol) | < -31 kcal/mol | The 2-8 bp of miRNA seed region strictly matched | Target gene log_2_ fold change > \|2.9\| |
| --- | --- | --- | --- | --- | --- | --- | --- |
| 1 | all_hsa-miR-1273h-5p | SESN3 | peroxidase | -36.1 | √ | √ | √ |
| 2 | all_hsa-miR-1273h-5p | DPAGT1 | glycosyltransferase | -23.6 |  |  |  |
| 3 | all_hsa-miR-1273h-5p | XRN1 | exoribonuclease | -31 | √ | √ |  |
| 4 | all_hsa-miR-1273h-5p | PFKFB2 | carbohydrate phosphatase | -35.9 | √ | √ |  |
| 5 | all_hsa-miR-330-5p | TCF12 | basic helix-loop-helix transcription factor; nucleic acid binding | -40.1 | √ | √ |  |
| 6 | all_hsa-miR-330-5p | ELF1 | nucleic acid binding; signaling molecule; winged helix/forkhead transcription factor | -37.9 | √ | √ |  |
| 7 | all_hsa-miR-342-5p | RNH1 | nucleic acid binding; transcription cofactor | -14.8 |  |  |  |
| 8 | all_hsa-miR-361-5p | MAP3K12 | - | -26.1 |  |  |  |
| 9 | all_hsa-miR-4470 | KAT6A | acetyltransferase; chromatin/chromatin-binding protein; zinc finger transcription factor | -21.1 |  |  |  |
| 10 | all_hsa-miR-6716-5p | IRF4 | nucleic acid binding; winged helix/forkhead transcription factor | -28.4 |  |  |  |
| 11 | all_hsa-miR-7854-3p | ZBTB21 | KRAB box transcription factor | -36.5 | √ |  |  |
| 12 | all_hsa-miR-874-5p | SUGP1 | mRNA splicing factor | -24.7 |  |  |  |
| 13 | all_hsa-miR-874-5p | ADAMTS2 | extracellular matrix glycoprotein; metalloprotease; serine protease inhibitor | -35.9 | √ | √ | √ |
| 14 | all_hsa-miR-874-5p | PLCH2 | calcium-binding protein; guanyl-nucleotide exchange factor; phospholipase; signaling molecule | -40.2 | √ |  |  |
| 15 | all_hsa-miR-874-5p | DUSP7 | - | -38.9 | √ |  |  |
| 16 | all_hsa-miR-874-5p | ERCC4 | endodeoxyribonuclease | -30.7 |  |  |  |
| 17 | all_hsa-miR-92b-5p | IREB2 | dehydratase; hydratase | -34.3 | √ | √ | √ |
| 18 | NovelmiRNA-25 | AMPD2 | deaminase | -31.2 | √ | √ | √ |
| 19 | NovelmiRNA-296 | MDN1 | - | -29 |  |  |  |
| 20 | NovelmiRNA-30 | ADAMTS10 | extracellular matrix glycoprotein; metalloprotease; serine protease inhibitor | -28 |  |  |  |
| 21 | NovelmiRNA-30 | DUSP16 | - | -26.7 |  |  |  |
| 22 | NovelmiRNA-30 | LONRF1 | ubiquitin-protein ligase | -25.3 |  |  |  |
| 23 | NovelmiRNA-30 | AHNAK | - | -27.7 |  |  |  |
| 24 | NovelmiRNA-30 | MGAT5 | glycosyltransferase | -33.8 | √ | √ |  |
| 25 | NovelmiRNA-376 | RNF14 | ubiquitin-protein ligase | -29.1 |  |  |  |
| 26 | NovelmiRNA-376 | ILKAP | kinase inhibitor; protein phosphatase | -27.2 |  |  |  |
| 27 | NovelmiRNA-376 | TP53I3 | dehydrogenase; reductase | -23.8 |  |  |  |
| 28 | NovelmiRNA-426 | SIK2 | non-receptor serine/threonine protein kinase | -33.9 | √ |  |  |
| 29 | NovelmiRNA-489 | NR4A2 | C4 zinc finger nuclear receptor | -28.2 |  |  |  |
| 30 | NovelmiRNA-72 | ELL2 | tight junction; transcription cofactor | -25.6 |  |  |  |
| 31 | NovelmiRNA-815 | SPEN | RNA binding protein | -28.8 |  |  |  |
| 32 | NovelmiRNA-862 | CBL | ligase | -38.5 | √ |  |  |
| 33 | NovelmiRNA-862 | NBEAL2 | - | -29.4 |  |  |  |
| 34 | NovelmiRNA-92 | TFIP11 | mRNA splicing factor | -31.3 | √ |  |  |
| 35 | NovelmiRNA-92 | CSNK1E | non-receptor serine/threonine protein kinase | -29.2 |  |  |  |

**Table S8. miRNAs differentially involved in the development and function of SLE.**

| Number | | | miRNA | | | mRNA | | Samples | | Function | | Reference |  |
| --- | --- | --- | --- | --- | --- | --- | --- | --- | --- | --- | --- | --- | --- |
| 1 | | | miR-19b and miR-20a | | | TF | | PBMC | | Increase TF expression, leading to hypercoagulability characteristics in these patients | | [1] |  |
| 2 | | | miR-126 | | | CD11a and CD70 | | PBMC | | Resulting in overactivation of T cells and B cells | | [2] |  |
| 3 | | | miRNA-126 | | | IFN-α and ISG56 | | PBMC | |  | | [3] |  |
| 4 | | | hsa-miR-939 | | | HSP27 and Brn3a | | PBLs | | HSP27, decreased expression of Brn3a, increased hsa-miR-939 levels, and was associated with selective anti-ENA(+) patients. HSP27 was negatively correlated with apoptosis | | [4] |  |
| 5 | | | miR-146a | | | ADL，CCL2，CXCL10 and STAT1 | | PBLs | | STAT1 acts as an enhancer of CCL2 and CXCL10 expression in the pathogenesis of SLE | | [5] |  |
| 6 | | | microRNA-30a | | | Lyn | | B cell | | Play an important role in B cell hyperactivity | | [6] |  |
| 7 | | | miR-125b | | | ETS1 and STAT3 | | PBMC | | Down-regulation of miR-125b, mainly in T cells, may contribute to the pathogenesis of SLE by regulating the expression of ETS1 and STAT3 genes. | | [7] |  |
| 8 | | | miR-145、miR-224 | | | STAT1、API5 | | T cell | |  | | [8] |  |
| 9 | | | miRNA 17-5p | | | E2F1 | | peripheral blood in children | |  | | [9] |  |
| 10 | | | miR-27a | | | NKG2D | | PBMCs | | The expression of miR-27a in PBMCs and NK cells enhanced the expression of NKG2D in SLE patients. In addition, ligands for NKG2D and ULBP2 were found to be down-regulated in PBMC of SLE patients. | | [10] |  |
| 11 | | | miR-371a-5p and let-7a-5p | | | TAGLN、AK4 | | SLE-iPSC | |  | | [11] |  |
| 12 | | | miR-410 | | | STAT3 | | peripheral blood lymphocyte | | Overexpression of miR-410 significantly reduced the expression of IL-10. In addition, miR-410 inhibits the transcriptional activity of STAT3 by directly binding the 3' UTR of STAT3 mRNA. In addition, silencing of STAT3 downregulates IL-10 expression in CD3+ T cells. | | [12] |  |
| 13 | | | miR-326 | | | Ets-1 | | Treg cells | | Increased expression of miR-326 in Treg cells of SLE patients may inhibit the involvement of Ets-1 in pathological processes of SLE. | | [13] |  |
| 14 | | | miR-1246 | | | EBF1 | | Peripheral blood B cells | | The expression of miR-1246 in B cells of SLE patients was significantly reduced. miR-1246 specifically targets EBF1 messenger RNA (mRNA) and regulates EBF1 expression through its interaction with its 3' untranslated region (3'-UTR). | | [14] |  |
| 15 | | | miR-155，miR-17 and miR-181b | | | AID and IFN-α | | PBMCs | | The expression levels of miR-155, miR-17, and miR-181b were negatively correlated with the AID and IFN-α mRNA expression levels in SLE patients. Luciferase reporter assays showed that miR-181b negatively regulates AID and IFN-α. | | [15] |  |
| 16 | | | miR-663 | | | TGF-β1 | | BMSCs | | miR-663 is a key mediator of SLE BMSC regulation and can be used as a new therapeutic target for the treatment of lupus. | | [16] |  |
| 17 | | | miR-29a | | | anti-β2GP1 | | Peripheral blood | | miR-29a positively correlated with anti-β levels of 2GP1 antibody | | [17] |  |
| 18 | | | miR-7 | | | PTEN | | PBMC | | PTEN is one of the target genes of miR-7. The expression of PTEN in B cells transfected with miR-7 precursor was decreased, which promoted the proliferation of B cells. miR-7 participates in the pathogenesis of SLE by regulating PTEN expression and B cell proliferation. | | [18] |  |
| 19 | | | miRNA-210 | | | HIF-1α | | PBMCs | | The expression of miR-210 in patients with SLE and pleuritis was significantly increased | | [19] |  |
| 20 | | | miR-31 | | | IL-2 and NF-At and RhoA | | T cells | | The expression of miR-31 in lupus T cells was significantly decreased and positively correlated with the expression of IL-2. Manipulation of miR-31 expression in lupus T cells restored IL-2 expression at both messenger RNA and protein levels. | | [20] |  |
| 21 | | | miR-125a | | | RANTES | | T cells | | MicroRNA-125a negatively regulates RANTES expression by targeting KLF13 in activated T cells. The low expression of miR-125a contributes to the elevated expression of RANTES in SLE. | | [21] |  |
| **Reference** | | |  | | |  | |  | |  | | |  |
| [1] | | | Teruel R, Perez-Sanchez C, Corral J, et al. Identification of mirnas as potential modulators of tissue factor expression in patients with systemic lupus erythematosus and antiphospholipid syndrome. J Thromb Haemost. 2011;9:1985-92. | | | | | | | |  |  |  |
| [2] | | | Zhao S, Wang Y, Liang Y, et al. Microrna-126 regulates DNA methylation in cd4+ t cells and contributes to systemic lupus erythematosus by targeting DNA methyltransferase 1. Arthritis Rheum. 2011;63:1376-86. | | | | | | | |  |  |  |
| [3] | | | Liu YJ, Fan WJ, Bai JZ. Microrna-126 expression and its mechanism of action in patients with systemic lupus erythematosus. Eur Rev Med Pharmacol Sci. 2015;19:3838-42. | | | | | | | |  |  |  |
| [4] | | | Rai R, Chauhan SK, Singh VV, et al. Heat shock protein 27 and its regulatory molecules express differentially in sle patients with distinct autoantibody profiles. Immunol Lett. 2015;164:25-32. | | | | | | | |  |  |  |
| [5] | | | Dominguez-Gutierrez PR, Ceribelli A, Satoh M, et al. Elevated signal transducers and activators of transcription 1 correlates with increased c-c motif chemokine ligand 2 and c-x-c motif chemokine 10 levels in peripheral blood of patients with systemic lupus erythematosus. Arthritis Res Ther. 2014;16:R20. | | | | | | | |  |  |  |
| [6] | | | Liu Y, Dong J, Mu R, et al. Microrna-30a promotes b cell hyperactivity in patients with systemic lupus erythematosus by direct interaction with lyn. Arthritis Rheum. 2013;65:1603-11. | | | | | | | |  |  |  |
| [7] | | | Luo X, Zhang L, Li M, et al. The role of mir-125b in t lymphocytes in the pathogenesis of systemic lupus erythematosus. Clin Exp Rheumatol .2013;31:263-71. | | | | | | | |  |  |  |
| [8] | | | Lu MC, Lai NS, Chen HC, et al. Decreased microrna(mir)-145 and increased mir-224 expression in t cells from patients with systemic lupus erythematosus involved in lupus immunopathogenesis. Clin Exp Immunol. 2013;171:91-9. | | | | | | | |  |  |  |
| [9] | | | Aboelenein HR, Salah S, Lashine YA, et al. Dual downregulation of microrna 17-5p and e2f1 transcriptional factor in pediatric systemic lupus erythematosus patients. Rheumatol Int. 2013;33:1333-8. | | | | | | | |  |  |  |
| [10] | | | Sourour SK, Aboelenein HR, Elemam NM, et al. Unraveling the expression of microrna-27a* & nkg2d in peripheral blood mononuclear cells and natural killer cells of pediatric systemic lupus erythematosus patients. Int J Rheum Dis. 2017;20:1237-46. | | | | | | | |  |  |  |
| [11] | | | Tang D, Chen Y, He H, et al. Integrated analysis of mrna, microrna and protein in systemic lupus erythematosus-specific induced pluripotent stem cells from urine. BMC Genomics. 2016;17:488. | | | | | | | |  |  |  |
| [12] | | | Liu D, Zhang N, Zhang X, et al. Mir-410 down-regulates the expression of interleukin-10 by targeting stat3 in the pathogenesis of systemic lupus erythematosus. Cell Physiol Biochem. 2016;39:303-15. | | | | | | | |  |  |  |
| [13] | | | Sun XG, Tao JH, Xiang N, et al. Negative correlation between mir-326 and ets-1 in regulatory t cells from new-onset sle patients. Inflammation. 2016;39:822-9. | | | | | | | |  |  |  |
| [14] | | | Luo S, Liu Y, Liang G, et al. The role of microrna-1246 in the regulation of b cell activation and the pathogenesis of systemic lupus erythematosus. Clin Epigenetics. 2015;7:24. | | | | | | | |  |  |  |
| [15] | | | Kaga H, Komatsuda A, Omokawa A, et al. Downregulated expression of mir-155, mir-17, and mir-181b, and upregulated expression of activation-induced cytidine deaminase and interferon-alpha in pbmcs from patients with sle. Mod Rheumatol. 2015;25:865-70. | | | | | | | |  |  |  |
| [16] | | | Geng L, Tang X, Zhou K, et al. Microrna-663 induces immune dysregulation by inhibiting tgf-beta1 production in bone marrow-derived mesenchymal stem cells in patients with systemic lupus erythematosus. Cell Mol Immunol. 2018. | | | | | | | |  |  |  |
| [17] | | | Li Y, Shen L, Ye Y, et al. The expression and clinical significance of mir-7 in peripheral blood of patients with systemic lupus erythematosus. Basic & Clinical Medicine. 2014;34:53-7. | | | | | | | |  |  |  |
| [18] | | | Xu L YL, Chen K, Huang Q, et al. Expression of mir-29a in plasma of patients with systemic lupus erythematosus and its clinical significance. Zhejiang Medical Journal. 2015;37:1565-9. | | | | | | | |  |  |  |
| [19] | | | Huang Q, Chen SS, Li J, et al. Mir-210 expression in pbmcs from patients with systemic lupus erythematosus and rheumatoid arthritis. Ir J Med Sci.2018;187:243-9. | | | | | | | |  |  |  |
| [20] | | | Fan W, Liang D, Tang Y, et al. Identification of microrna-31 as a novel regulator contributing to impaired interleukin-2 production in t cells from patients with systemic lupus erythematosus. Arthritis Rheum. 2012;64:3715-25. | | | | | | | |  |  |  |
| [21] | | | Zhao X, Tang Y, Qu B, et al. Microrna-125a contributes to elevated inflammatory chemokine rantes levels via targeting klf13 in systemic lupus erythematosus. Arthritis Rheum. 2010;62:3425-35. | | | | | | | |  |  |  |
